# Enhancing learning and retention with distinctive virtual reality environments and mental context reinstatement

**DOI:** 10.1038/s41539-022-00147-6

**Published:** 2022-12-08

**Authors:** Joey Ka-Yee Essoe, Nicco Reggente, Ai Aileen Ohno, Younji Hera Baek, John Dell’Italia, Jesse Rissman

**Affiliations:** 1 Center for OCD, Anxiety, and Related Disorders for Children, Division of Child and Adolescent Psychiatry, Department of Psychiatry and Behavioral Sciences, The Johns Hopkins University School of Medicine, Baltimore, MD 21205 USA; 2 Department of Psychology, University of California, Los Angeles, CA 90095 USA; 3 Institute for Advanced Consciousness Studies, Santa Monica, CA 90403 USA; 4 School of Medicine, California University of Science and Medicine, Colton, CA 92324 USA; 5 Division of Psychology, Communication, and Human Neuroscience, School of Health Sciences, Faculty of Biology, Medicine and Health, University of Manchester, Manchester, M13 9PL UK; 6 Birmingham Veterans Affairs, Birmingham, AL 35233 USA; 7 Department of Psychiatry & Biobehavioral Sciences, University of California, Los Angeles, CA 90095 USA; 8 Brain Research Institute, University of California, Los Angeles, CA 90095 USA; 9 Integrative Center for Learning and Memory, University of California, Los Angeles, CA 90095 USA

**Keywords:** Learning and memory, Language

## Abstract

Memory is inherently context-dependent: internal and environmental cues become bound to learnt information, and the later absence of these cues can impair recall. Here, we developed an approach to leverage context-dependence to optimise learning of challenging, interference-prone material. While navigating through desktop virtual reality (VR) contexts, participants learnt 80 foreign words in two phonetically similar languages. Those participants who learnt each language in its own unique context showed reduced interference and improved one-week retention (92%), relative to those who learnt the languages in the same context (76%)—however, this advantage was only apparent if participants subjectively experienced VR-based contexts as “real” environments. A follow-up fMRI experiment confirmed that reinstatement of brain activity patterns associated with the original encoding context during word retrieval was associated with improved recall performance. These findings establish that context-dependence can be harnessed with VR to optimise learning and showcase the important role of mental context reinstatement.

## Introduction

Considerable research has documented that human memory is inherently context-dependent^1,2^. During learning, contextual cues—whether environmental (e.g., a specific room) or internal (e.g., an emotional state)—become bound to the information being encoded. Although some of these cues may be relevant to the to-be-learnt materials, many will be seemingly irrelevant. Despite their relevance, the later presence of these same contextual cues can facilitate memory recall, whereas their absence can hinder recall^3^. Perhaps the most iconic example of this effect is Godden & Baddeley’s^4^ demonstration that scuba divers were better able to recall words that they had studied underwater when tested underwater, and better able to recall words studied on land when tested on land, but impaired when these study and test contexts were mismatched. Context effects can be observed with far less dramatic environmental changes (e.g., being tested in a different room^5^, or in a more quiet/noisy environment^6^), are most robust when memory is probed with recall rather than recognition tests^1,7^.

One situation where context effects can be particularly impactful for learning is when multiple sets of information are studied in close temporal proximity. When the to-be-learnt content is similar across these sets, the build-up of interference can make it difficult to maintain clear mental representations of each set and cause confusion between the sets. For instance, reading two conceptually similar scientific papers within the same hour may lead one to mentally misattribute a finding of one paper to another. Likewise, while traveling to a place where two phonetically similar languages are spoken, it might be challenging to keep vocabulary items in these two languages appropriately compartmentalised in one’s memory if they are studied on the same plane flight. Some research has shown that learning each information set in its own distinctive context can improve recall by reducing this type of interference^8,9^. Specifically, a distinctive context provides unique cues that will become bound to items from a given information set. This supports learners’ abilities to maintain separate mental representations, reducing interference between the sets. This context-induced benefit increases in magnitude when the contexts are more distinctive, and when fewer items are affiliated with each context^1,8–10^.

Although distinctive learning contexts have the potential to reduce interference, they run the risk of creating context-dependent associations that could hinder later recall under circumstances where those contextual cues are no longer present. Whenever individuals have the luxury of studying information and repeatedly taking practice tests on that information in a single context, they may acquire the information quickly and perform quite well without realising the extent to which they are using the contextual cues as a “crutch” to facilitate learning and retrieval^9,11^. Only when later struggling to recall the information in a new context—such as a foreign traveller trying to use vocabulary that had only ever been practiced in a classroom setting—does their reliance on this contextual crutch become apparent. In most real-world settings, it is impossible or impractical for learners to physically return to the original encoding context as a means to gain access to helpful retrieval cues. Fortunately, mental reinstatement—the act of vividly imagining oneself in the original encoding environment—presents one solution to promote information transfer across contexts. Indeed, mental reinstatement can be nearly as effective as physically returning to the learning context^2,12^. Thus context change-induced forgetting may be mitigated by mentally “returning” to the learning context during recall.

Learning protocols that harness the beneficial aspects of context-dependence while ameliorating the deleterious effects are likely to yield the best outcomes. How best to achieve this balance thus remains an active and important area of research. Designing and controlling distinct contexts in practice is challenging, for experimenters and learners alike. Manipulating one’s physical context can influence learning and recall, but doing so can be costly, time-consuming, and difficult to control. Background images^13^ and videos^14^ have been used as contexts in an effort to increase experimental control. While these can serve as proximate contextual cues in experiments, they do not allow navigation or immersion like real-world contexts, and thus ecological validity suffers^15^.

Virtual reality (VR) offers a powerful means to create immersive learning environments that are highly distinctive and well-controlled, in order to examine and exploit context-based memory modulation^15,16^. Indeed, one recent study used two distinctive VR-based contexts—one underwater and one on the surface of Mars^17^—to conceptually replicate Godden & Baddeley’s classic finding of context-dependent recall. When using VR environments as contexts, it is valuable to measure participants’ sense of *presence*^18–21^, which refers to their sense of experiencing a VR-based environment as a place that one has actually inhabited, rather than something that one was merely watching passively (e.g., “I feel like I am in this space station, walking around,” *vs* “I am watching this space station on a screen while sitting in a lab.”). If an individual does not perceive VR-based contexts as actual environments, then these contexts may have little or no effect on memory outcomes because the “contexts” themselves would not be subjectively valid.

Here, we aimed to leverage the benefits of context-dependence to enhance learning and retention. We chose to focus on foreign vocabulary learning as it is a domain of practical value to many people, while also being a paradigmatic paired associate learning task. To rigorously test this approach, we selected learning material to maximise potential interference and used a challenging recall test. English-speaking participants learnt the meanings and pronunciations of 80 foreign words from two phonetically similar Bantu languages: Swahili and Chinyanja. During testing, participants were prompted to verbally pronounce foreign words when cued with their English translations (note that this is far more difficult than being cued with the foreign word and recalling the English translation^22^).

Two custom, first-person desktop VR environments served as contexts, which enabled maximal experimental control over the learning contexts and subsequent guided mental reinstatement. First, we investigated whether contextual support could improve learning outcomes by reducing interference and promoting transfer. To this end, participants were randomly assigned to one of two groups: a *single-context* (*n* = 24) group that learnt both languages in a single VR context, and a *dual-context* (*n* = 24) group that learnt each language in its own unique VR context. We hypothesised that dual-context participants would be better able to keep track of which translations went with which language and thus would show fewer intrusions (i.e., producing the Chinyanja translation of a word when cued to recall the Swahili translation), and greater long-term retention (as measured on a surprise recall test conducted one week later). Moreover, we predicted that the magnitude of these context effects might be contingent on whether participants subjectively experienced the VR-based contexts as actual environments they had inhabited (i.e., did they have a strong sense of presence?). Thus, a 10-item presence scale (range 1-5) from a prior study was used to measure the degree to which participants felt “as one” with their first-person avatar and experienced the VR as real environments^19^. To assess the role of mental context reinstatement, our paradigm explicitly cued participants to imagine themselves in a specified place prior to each vocabulary recall trial. This allowed us to measure the impact of context reinstatement congruency (i.e., whether they reinstated the same or different context in which they had learnt a given language) on recall performance. Finally, to further explicate a potential mechanism for contextually supported recall, we examined a separate group of dual-context participants (*n* = 22) during recall, using functional magnetic resonance imaging (fMRI) to provide a neural index of context-specific reinstatement on each retrieval trial^23,24^. We hypothesised that elevated reinstatement of brain activity patterns linked to the original encoding context would enhance the likelihood that participants would be able to successfully recall the cued foreign vocabulary item. Given the universal desire to develop protocols for memory enhancement across disciplines, this investigation holds considerable promise for fields such as cognitive research, pedagogy, and psychotherapies that involve therapeutic skill learning.

## Results

### Initial learning: contextual crutch and desirable difficulties

**Fig. 1:**
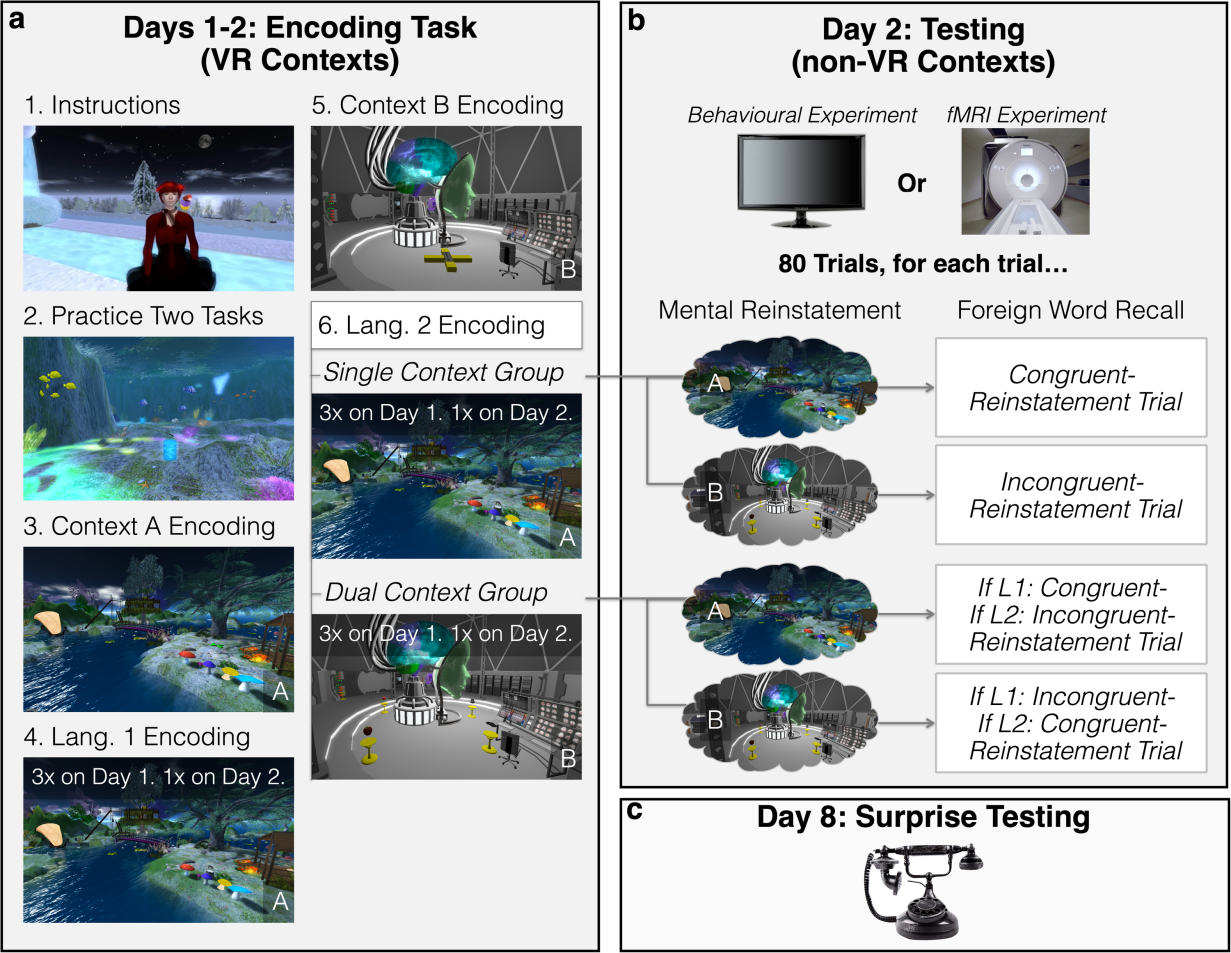
Experimental design. **a** Encoding tasks in VR-based contexts across Days 1 and 2. a1, In an underwater practice context, participants learnt VR navigation and received tasks instructions from “the teacher.” a2, Task Practice (under experimenter supervision). a3, Context A Encoding. In each of Context A’s nine named “rooms”, participants stood on a location marker and performed two clock-wise rotations (720°), while imagining themselves as tourists who forgot their camera, trying to remember what it felt like to be there. a4, Language 1 Encoding. Participants remained in Context A to encode Language 1 (Rounds 1–3, 40 words per round). a5, Context B Encoding. a6, Language 2 Encoding (Rounds 1–3). All participants experienced the same procedures except for the context in which Language 2 was encoded. *Single-context* participants returned to Context A to encode Language 2, while *dual-context* participants remained in Context B to encode Language 2. On Day 2 participants performed Rounds 4 of Language 1 and Language 2 Encoding. **b** Day 2: short-delay recall (T4). After a short delay, participants were tested outside of the VR contexts, in the laboratory or MRI scanner. In each of 80 trials, participants first mentally reinstated an auditorily cued room from one context before recalling the foreign translation of a cued word. In *congruent reinstatement* trials, the mentally reinstated room was the learning context of the cued word. In *incongruent reinstatement* trials, the mentally reinstated room was in the opposite context. **c** Day 8: one-week-delayed recall (T5). Participants were telephoned, ostensibly for an interview; experimenters then cued recall for all 80 foreign words. Image attribution: The VR environments and content depicted here were created by J.K.-Y.E or by Forde Davidson as commissioned by the research team, or were from the OpenSim community shared under the Creative Commons 0 License. The image of the telephone and computer monitor were modified from public domain images, and the image of the MRI scanner was provided by the UCLA Brain Mapping Center.

**Fig. 2:**
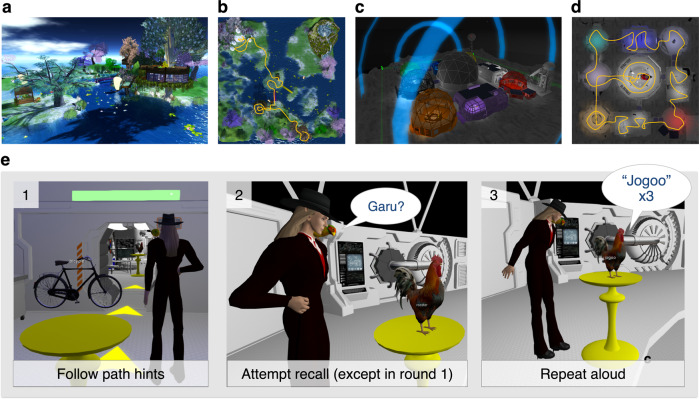
Distinctive VR-based contexts and language encoding task. Two custom-built VR-based contexts were used in this study. **a** “Fairyland Garden” was a fantasy-fiction inspired context that was bright, verdant, visually open, with lakes and wooden rooms opened to the outdoors. **b** Fairyland Garden’s predetermined path used in language encoding. This path’s hints were bright green footsteps; its pedestals tree stumps. **c** “Moon Base” was a science-fiction inspired context that was dark, rocky, closed-in, with narrow hallways and artificially coloured metallic rooms, and participants were confined indoors at all times. **d** Moon Base’s predetermined path used in language encoding. This path’s hints were bright yellow arrows; its pedestals yellow stands as shown in 2e. **e** Language encoding task. In each round of language encoding, participants interacted with 40 concrete objects representing each of the foreign words (e.g., a rooster), organised along a predetermined path. The VR environments were experienced through a first-person perspective (a visible avatar is only present in this figure for illustrative purposes). e1, Participants followed visual hints (e.g., arrows) to an object; these hints were transient and disappeared after use. After arriving at the object, participants first verbally say its English name (e.g., “rooster”), printed in floating text above the object. During Round 1 of each language, participants then ‘clicked’ the object. e2, During Rounds 2–4, participants first attempted to verbally recall the foreign words (T1-T3) before clicking the object. e3, When the object was clicked, participants would hear the foreign translation (e.g., Swahili word “jogoo,” meaning rooster) three times. They were to repeat aloud after it each time. Then they clicked the object’s pedestal to reveal transient path hints to the next object. Image attribution: The VR environments and content depicted here were created by J.K.-Y.E or by Forde Davidson as commissioned by the research team, or were from the OpenSim community shared under the Creative Commons 0 License.

Across two consecutive days, participants encoded a total of 80 foreign vocabulary items in two languages, in one learn-only round (Round 1), followed by three test-learn cycles (Rounds 2–4, retrieval attempts during these tests were scored as recall data for *Times 1-3; T1-T3*. See Fig. 1, Fig. 2e, Methods, and Supplementary Video 1). They learnt 10 words in Swahili only, 10 in Chinyanja only, and 30 words in both languages. To induce contextual crutch effects, test-learn cycles occurred within the learning context(s) as participants navigated along a predetermined path (Fig. 2a–d). To further bolster initial learning we integrate a “desirable difficulties” technique^25^ called expanding retrieval practice, in which the time interval between successive learning and testing opportunities progressively increased^26^. Differences between the single-context and dual-context groups were not expected to emerge during the initial learning stage, as the magnitude of context effects has been shown to increase with the length of the retention interval^1^.

**Fig. 3:**
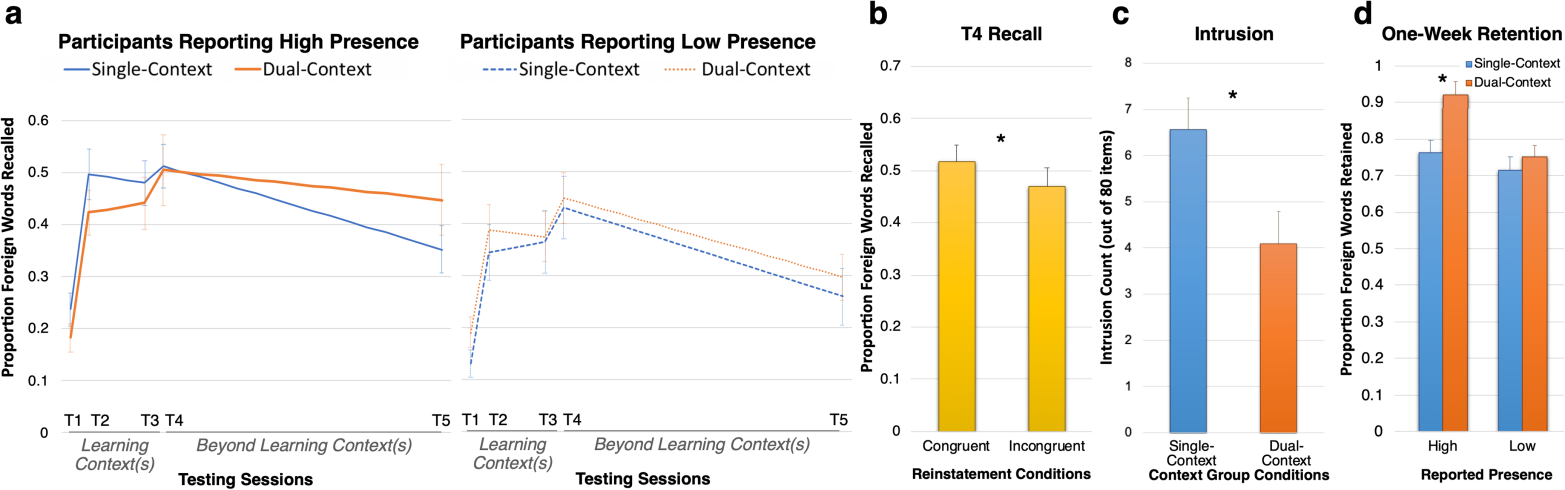
Behavioural experiment results. **a** overall recall performance, split by context group and presence. **b** Main effect of mental reinstatement on T4 recall. **c** Main effect of context group condition on intrusions. **d** Interactions of context group and presence in one-week retention. * denotes statistical significance, error bars denote standard error of the mean.

Across groups, participants recalled 42% (±17%) of the 80 foreign words after two exposures (T2); note that each “exposure” refers to encountering an object and hearing and repeating back its translation three times in rapid succession (Fig. 3). This learning rate was considerably higher than expectations (22–26%) set based on a previous study that used similar learning material (42 Swahili-English word pairs; no secondary foreign language was learnt in that study), but did not employ distinctive learning contexts (see Supplementary Discussion: D2 for additional discussion)^27^. After the third exposure to the foreign words, our participants were not tested until the following day (T3), and yet their recall performance remained robust at 42% (±17%). As expected, no group differences emerged during the initial learning stage (*p* > 0.05).

### Transfer and mental reinstatement

**Fig. 4:**
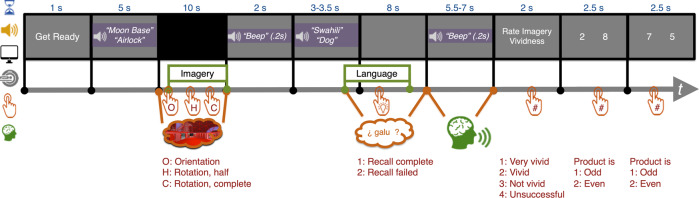
Short-delay non-VR test. An example trial of the short-delay non-VR test. Each trial consisted of the following periods: Mental reinstatement, language recall, imagery vividness rating, and two arithmetic questions (which served as an active baseline period between trials). The words “Get Ready” appeared to indicate the start of each trial. Mental Reinstatement: Participants heard via headphone the name a room they had visited (e.g., “Moon Base: Airlock”). Then the screen turns black, cuing participants to close their eyes and mentally “place” themselves back in that room. They pressed Button 1 to indicate that they had successfully “arrived” and oriented themselves. Then they mentally performed the same rotations they had done in the context encoding task (Figs. 1a.3, 1a.5), while pushing Buttons 2 and 3 to indicate their mental reinstatement progress until they heard a beep. In the fMRI experiment, brain activity patterns related to mental imagery were extracted for the period between the Button 1 press and the beep. Language Recall: Participants heard the language recall cue (e.g., “Swahili: Dog”). Participants began to covertly retrieve the foreign word and made a button-press to indicate success or failure of retrieval; they then continued thinking about that word until they heard a beep. Upon the beep, they verbally pronounced the foreign word, or the portion of it they could recall. In the fMRI experiment, brain activity patterns related to language recall were extracted from the 6 s after the audio cue offset. Imagery Rating: Participants rated how vivid the previous mental reinstatement had been. These ratings were later used for trial exclusion for analyses involving mental reinstatement. Arithmetic Questions: At the end of each trial, participants answered two simple arithmetic questions. Each involved a display of two single-digit integers, and they were to press Button 1 if the product of these numbers was odd, and Button 2 if even.

Transfer was measured by recall during a non-VR test (T4), which was the first test that occurred outside of the learning context. Across conditions, participants recalled 48% (±18%) in T4. A controlled mental reinstatement protocol was employed to maximise consistency across participants and across experiments (Fig. 4; see Methods). On each trial, participants were first cued to mentally reinstate a specific area within a given learning context (e.g., “Moon Base: Airlock”). Then, they were prompted by audio cues (e.g., “Swahili: dog”) to attempt to covertly retrieve the appropriate foreign translation, and finally a beep sound cued them to verbally pronounce the word. Two mental reinstatement conditions were employed: congruent reinstatement (when the original learning context of the to-be-recalled word was mentally reinstated) and incongruent reinstatement (when a different context was mentally reinstated). During T4, congruent mental reinstatement trials exhibited significantly greater recall (52% ± 18%) than incongruent reinstatement trials (47% ± 19%), RM-ANOVA, *p* = 0.009, *η*_*p*_^*2*^ = 0.31; Fig. 3b; see Supplementary Note 1: A2, A3). This demonstrated that when recalling in a new context, transfer is enhanced when the learning context is mentally reinstated. This effect did not interact with context-group membership, suggesting that even those participants who learnt both languages in a single context still benefitted when prompted to mentally reinstate that context relative to when they reinstated a context in which neither language had been learnt.

### Interference reduction

Interference was measured by intrusions from the opposite language (i.e., producing the Chinyanja translation of a word when cued to recall the Swahili translation, or vice versa), as these indicate a failure to maintain clear and distinctive representations between the two languages. While the intrusion count was generally low (less than 10 items out of 80), dual-context participants exhibited 38% fewer intrusions (4.09 ± 4.82) than the single-context (6.57 ± 4.69) participants (Fig. 3c; RM-ANOVA, *p* = 0.014, *η*_*p*_^*2*^ = 0.13; see Supplementary Note 1: A3). This suggests that learning each language in its own distinctive context helped participants to maintain better separated mental representations and reduced interference.

### One-week retention

A surprise memory test (T5; Fig. 1d) was conducted via telephone one-week after T4. In a pre-scheduled “follow-up interview,” experimenters asked participants several interview questions and then began to conduct T5 (e.g., “How do you say ‘cherry’ in Chinyanja?”). Retention score was the percentage of information that survived the one-week delay interval, after it had been previously recalled in T4 (i.e., words that were not successfully recalled in T4 were excluded, see Methods). Furthermore, as the context manipulation was conducted via VR, presence (one’s sense of inhabiting a VR-based context as a real location) was entered into the analyses as a factor—if participants did not experience the VR environments as real contexts, then the context manipulation should have little to no effect.

Results showed that amongst participants who reported high presence (based on a mean split of presence scores, see Supplementary Table 2), the dual-context group exhibited a striking 92% (±7%) one-week retention rate, which was significantly higher than 76% (±12%) retention rate exhibited by the single-context group (Fig. 3d; RM-ANOVA interaction, *p* = 0.03, *η*_*p*_^*2*^ = 0.11; simple main effect, *p* = 0.002; see Supplementary Note 1: A4). Single- and dual-context participants who reported low presence did not perform differently on one-week retention (simple main effect for low-presence participants, *p* = 0.47), nor did they differ from single-context participants reporting high presence (all contrasts *p* > 0.05). Collectively, these results demonstrate that contextual support from unique contexts dramatically enhanced one-week retention, but only when participants subjectively perceived the contexts as actual environments they had inhabited.

### Neural correlates of contextually supported recall

To further investigate the mechanisms by which distinctive learning contexts can later be brought back to mind to support the recall of foreign vocabulary items, we conducted a follow-up fMRI experiment. We recruited a separate group of participants (*n* = 23; analyses included *n* = 22; see Methods) and assigned them all to the dual-context learning condition, since our goal was to measure context-specific reactivation on individual recall trials so as to characterise the behavioural advantage afforded by such reactivation. Given resource constraints, it was not possible for us to scan a separate group of single-context participants, nor would fMRI data from such participants be especially useful for our primary research question.

**Fig. 5:**
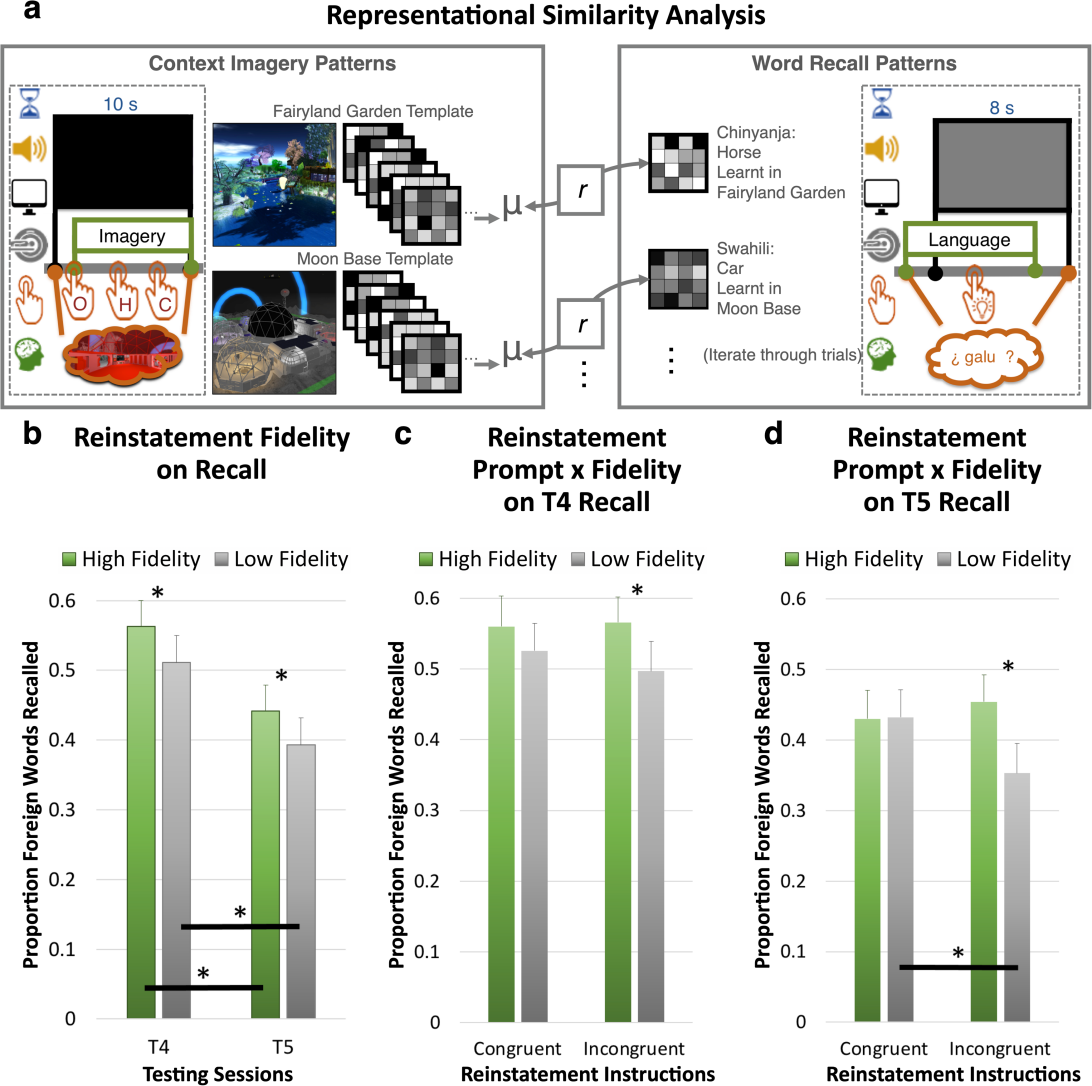
fMRI experiment: representational similarity analysis procedure and results. After feature selection, fMRI activity patterns from each participant’s top 2000 voxels were used in a within-subject representational similarity analysis (RSA); RSA output was used to analyse verbal recall data. **a** RSA computed the correlations between activity patterns for each word during covert word recall (right) and the context template (left) of the word’s original learning context. The context template was an average of all the imagery patterns for a given context. The resulting correlation values were then used to divide recall trials into high fidelity vs low fidelity reinstatement trials, and verbal recall results were examined for each trial type. The effects of reinstatement prompt (congruent vs. incongruent) and/or reinstatement fidelity (high vs. low) on recall are plotted respectively for: (**b**), all non-VR tests (T4 and T5; collapsed across reinstatement prompt conditions), (**c**), short-delay non-VR test (T4), and (**d**), one-week-delayed non-VR test (T5). * denotes statistical significance for pairwise tests; see main text for description of interaction effects. Image attribution: The VR environments depicted here were created by J.K.-Y.E. or by Forde Davidson as commissioned by the research team, or were from the OpenSim community shared under the Creative Commons 0 License. The icons used were either created by J.K.-Y.E. or were modified from stock icons in MS PowerPoint or public domain.

The use of verbal material separated the sensory modalities between contexts (visuospatial) and memoranda (verbal/auditory), allowing us to disentangle the neural correlates of contextual support from the memory retrieval itself. First, a whole-brain Searchlight Multi-Voxel Pattern Analysis (Supplementary Fig. 1; SL-MVPA) identified brain regions whose local fMRI activity patterns could most accurately discriminate between the two contexts during the mental reinstatement period. Each participant’s resulting searchlight map was thresholded to create an individualised binary mask, indicating which 2000 voxels would be used for the subsequent steps. Because the particular voxels selected for each participant will differ, we are unable to make claims about how individual brain regions contributed to our analyses. However, in an effort to provide a coarse portrait of which regions’ local activity patterns tended to be most able to facilitate context decoding, the group mean of the searchlight map is visualised in Supplementary Fig. 2 and shows that peak decoding was observed in bilateral visual association regions (superior lateral occipital cortex, ventral occipito-temporal cortex, fusiform gyrus), medial parietal regions (precuneus, posterior cingulate cortex), lateral parietal regions (intraparietal sulcus and superior parietal lobule), and the left inferior frontal sulcus. Second, a brain-response pattern was derived within this mask for each of the two learning contexts (Fig. 5a; context template). Third, a Representational Similarity Analysis (Fig. 5a; RSA) produced a similarity score between (1) the brain patterns during covert retrieval of each word and (2) the context template of the learning context of that word. This RSA score provided an objective, quantitative measure for mental contextual reinstatement during verbal recall for each individual trial, which we will refer to as its “representational fidelity.” Fourth, the verbal recall scores of words with high *vs* low representational fidelity (mean-split within-subject) were compared—which allowed us to examine whether trials with greater evidence for contextually supported retrieval enjoyed a behavioural performance advantage relative to those with less evidence for contextually supported retrieval.

A main effect of representational fidelity was observed (RM-ANOVA, *F*(1, 21) = 13.712, *p* = 0.001, *η*_*p*_^*2*^ = 0.395; see Supplementary Note 2), where high representational fidelity trials (0.50 ± 0.17) were associated with 5% higher recall than low representational fidelity trials (0.45 ± 0.18), collapsing across the short-delay test (T4) and one-week-delayed test (T5). When broken down by Times (Fig. 5b), the effect of representational fidelity was significant at both T4 (RM-ANOVA, *F*(1, 21) = 8.60, *p* = 0.008, *η*_*p*_^*2*^ = 0.29; High = 0.56 ± 0.19; Low = 0.51 ± 0.20) and T5 (RM-ANOVA, *F*(1, 21) = 8.53, *p* = 0.008, *η*_*p*_^*2*^ = 0.29; High = 0.44 ± 0.19; Low = 0.39 ± 0.20) in follow-up analyses. Furthermore, a significant interaction between reinstatement prompt and representational fidelity was observed across T4 and T5 (RM-ANOVA, *F*(1, 21) = 6.59, *p* = 0.02, *η*_*p*_^*2*^ = 0.24; not shown). This examined how recall performance was impacted by the relationship between representational fidelity and the reinstatement prompt at the beginning of each trial (i.e., whether participants were cued to recall a room in a context congruent or incongruent with the language that was about to be probed). Follow-up analyses revealed that this interaction was driven by T5 one-week delayed recall (*simple interaction: p* = 0.006; Fig. 5d), and not T4 short-delay recall (*p* > 0.05; Fig. 5c). After incongruent mental reinstatement, if representational fidelity had been high during T4 recall, participants enjoyed a 10.1% advantage one week later (0.45 ± 0.19) as compared to if representational fidelity had been low (0.35 ± 0.20). This effect was absent in the trials preceded by congruent mental reinstatement, and recall was still high for both conditions (both 0.43 ± 0.20).

These findings indicated that we were able to quantify contextual support via mental reinstatement—by identifying neural representations of the two learning contexts and measuring their expression during each covert word retrieval attempt. Overall, we found a striking relationship between trial-specific evidence of context reinstatement fidelity and the likelihood of successfully recalling the cued word in the specified language on that trial. The behavioural advantage of high-fidelity reinstatement was not only present in the immediate term (T4 recall) but also persisted after a one-week delay (T5 recall). That this advantage was most apparent during incongruent reinstatement trials indicates that as long as participants were able to reinstate the original learning context during the word recall phase (despite having been prompted to imagine a different context several seconds earlier) they could minimise the potential disadvantage of this contextual incongruency.

## Discussion

By using distinctive virtual reality environments to provide rich contextual support, our behavioural protocol facilitated robust learning of highly challenging material—foreign vocabulary in two phonetically similar languages—while ameliorating the negative effects of context-dependence via “desirable difficulties” and mental reinstatement. These memorable contexts could later serve as retrieval cues when mentally reinstated during recall. After only four learning sessions, participants were able to recall nearly half of the 80 foreign words they had studied, and they showed relatively little forgetting after one week (up to 92% retention). Importantly, the knowledge acquired within the VR-based contexts transferred well to support recall in non-VR settings (i.e., a laboratory testing room, an MRI scanner, and a surprise telephone test), despite the fact that the learning contexts shared relatively few cues with real-world environments. In so doing, we leveraged the benefits of the “contextual crutch” phenomenon whereby rapid acquisition was facilitated by repeatedly learning and testing in the same context while mitigating the deficits of transfer and retention that typically accompany this occurrence (See Supplementary Discussion: D3)^1,11,28^.

Our results provide evidence that contextual support optimises language learning in a manner that leads to high retention—but only when three critical conditions are met: First, participants must subjectively experience the VR-based contexts as actual environments that they feel like they are physically inhabiting during learning (i.e., they must report a high sense of presence). Second, a unique context must support the learning of each language. A high degree of presence, on its own, was insufficient to enhance retention for those participants in the single-context group who learnt the two languages in the same VR-based context. Only those participants assigned to the dual-context group—and who exhibited high presence during learning—showed superior retention of the material at the long-delayed test conducted one-week later. These high-presence dual-context participants were subjectively learning the two languages while actively navigating through two very different places, whereas low-presence participants presumably felt like they were learning both languages while sitting in a laboratory testing room. Third, benefits to memory recall must be evaluated after a long delay. Although dual-context participants did show fewer intrusions of the incorrect language translations (e.g., producing the Swahili translation when cued to recall the Chinyanja translation) at the immediate non-VR test (i.e., T4 on Day 2), they didn’t show an overall improvement in recall performance on this test. The dual-context participants’ advantage only emerged after the passage of one week’s time (i.e., T5 on Day 8). This finding illustrates that learning the two languages in two distinctive contexts can protect against forgetting, but only if participants felt highly present within the contexts. That the benefit was only observed after a long delay is consistent with previous reports that context-dependent effects tend to increase with longer retention intervals^1,29^. This may be due to the fact that that at shorter retention intervals a greater number of internal contextual cues (e.g., moods, levels of hunger or fatigue, private thoughts, etc.) may match those present during learning, thus outshining the effects of environmental context. Because we only assessed memory immediately after learning and at a one-week delay, we are unable to draw precise conclusions about the time course of the dual-context advantage. It is possible that the advantage could have emerged sooner (e.g., on Day 3 after one additional night of sleep), and it is also possible the magnitude of the effect could have grown even larger over time (e.g., if we waited two weeks before conducting the surprise memory test).

One critical attribute of our task design was the experimentally cued mental reinstatement of a specific environmental context prior to each vocabulary recall trial. This manipulation gave us precise experimental control over participants’ mental content immediately preceding each retrieval attempt. The cued context could either be congruent with the information the participant was about to be tested on (i.e., imagining themselves in the exact same ‘room’ where they had learnt that vocabulary item) or it could be incongruent (i.e., imagining themselves in a different ‘room’ from a completely different environment). Consistent with prior evidence for the benefits of mental reinstatement^2,12^, we found that imagery-based reinstatement of the congruent learning context enabled better recall in the short-delay non-VR test (i.e., T4).

In order to gain further insight into the impact of context reinstatement, we devised a follow-up experiment that used fMRI to measure neural correlates of context representations. This provided an objective index of the degree to which learning contexts were mentally reinstated during the language recall period of each trial. Unlike the behavioural experiment, the fMRI experiment enabled us to quantify mental reinstatement without relying on inferring mental reinstatement based on task instructions and participants’ subjective reports, nor to rely on the assumption that the reinstatement state would linger from the mental reinstatement period into the language recall period. Our fMRI experiment revealed evidence for contextually-supported retrieval of verbal materials. The results demonstrated that increased brain pattern similarity to the original learning context during covert verbal retrieval was associated with more successful recall performance. Trials with high reinstatement fidelity scores yielded short-delay recall performance (i.e., recall that took place seconds later) that was 5% higher than trials with low reinstatement fidelity scores. These high-fidelity reinstatement trials continued to enjoy the 5% recall advantage when memory was again tested one week later. This result expands upon a recent demonstration that context-specific fMRI activity patterns, induced through a closed-loop neurofeedback procedure, could facilitate verbal recall when the reinstated context was congruent with the learning context^30^.

When we examined the joint effects of mental reinstatement prompts and representational fidelity, we noted an interesting pattern. While high-fidelity mental reinstatement during recall improved short-delay recall regardless of pre-recall reinstatement prompts, after a one-week delay (T5) this advantage only appeared for words that had been paired with an incongruent pre-recall reinstatement prompt during T4. Thus, instructions to imagine oneself in a context that, just moments later, turns out to be incongruent with the learning context of the prompted language will serve to diminish the one-week retention of that word unless the participant manages to counteract this initial miscue and engage in high-fidelity reinstatement of the original learning context during word recall. In this sense, the act of overcoming incongruently cued context reinstatement by rapidly bringing the correct context back to mind may be considered a “desirable difficulty,”^25^ given its ability to promote one-week retention.

Although our study did not systematically compare the influence of spatial contexts with other aspects of event representation, our findings are consistent with the notion that spatial context is crucial in event representations. There is growing evidence that spatial context is possibly a dominant attribute over and above other episodic details (e.g., objects and persons)^31,32^. Intracranial electroencephalographic recordings from human hippocampus show that spatial context information is often reactivated earliest in the retrieval process and guides recall of items learnt in that context^33^. When recalling short stories, spatial cues lead to quicker and more detailed memories about events^34^. In a VR learning paradigm based on the Method of Loci mnemonic techniques, we previously demonstrated that memory for the spatial layout of VR environments is correlated with participants’ ability to recall words learnt in those environments^35^. Even though the contexts used in the present study’s foreign vocabulary learning task bore no direct relevance to the verbal content being learnt, these richly detailed virtual environments provided a consequential scaffolding that helped mitigate potential interference^36^ and provided memorable spatial cues that learners could later think back to when attempting word recall. While we did not directly test for this, the ability of our participants to actively navigate through the contexts during learning was likely an important determinant of the contextual effects we observed. One prior study investigating context-dependency used VR environments as passively presented backgrounds during word learning and found no impact of context reinstatement on behaviour^37,38^. Although there were other critical differences between our respective paradigms, this suggests that investigation of context effects will benefit when contexts are experienced in a more ecologically valid manner—such as the navigable, interactive desktop VR used here. When such contexts are experienced in VR, our results expand upon prior work emphasizing the importance of high presence in mediating the mnemonic benefits^37^. More broadly, our results showcase the critical importance of context in learning and bolster recent calls for cognitive neuroscientists to move beyond the study of isolated decontextualised stimuli^39^.

Presence, in addition to enabling virtual environments to serve as contexts for context-dependent memory effects, may be contributing to enhance learning in its own right. The recent Cognitive Affective Theory of Immersive Learning (CAMIL)^40^ would predict that VR experiences that induce a sense of presence can increase learner interest and intrinsic motivation, which in turn generates greater learner efforts and willingness to attend the task, thereby facilitating learning and recall. Indeed, engaging learning environments using head-mounted display (HMD)-based VR, and generative learning activities therein, have been found to lead to better transfer^41,42^. Although we did not quantitatively examine our participants’ interest, intrinsic motivation, or engagement, these advantageous internal contexts during our desktop VR-based learning tasks may have contributed to our participants recalling 42% of the 80 foreign words after only two exposures, considerably higher than a previous non-VR study (22–26%) that used arguably easier to-be-learnt material without distinctive learning contexts^27^. Furthermore, CAMIL would posit that if the VR contexts were more meaningful and relevant to the to-be-learnt items, the learning enhancement effects would be greater still due to an increased sense of presence and agency.

Our study has several limitations that should be addressed in future work. In an effort to gain greater experimental control, we elected to cue mental reinstatement of a specific context immediately prior to each foreign word recall prompt. While this manipulation allowed us to examine the effects of reinstatement congruency and facilitated our effort to create context-specific brain activity templates, it prevented us from knowing how our participants would have performed—and to what degree neural reinstatement would have predicted their performance—had we not invoked any explicit reinstatement instructions. Also, our use of fMRI was focused on using neural measures to index putative mental states, which we could then relate to behaviour. Although our whole-brain multivariate pattern analysis approach afforded us enhanced power in our ability to measure context reactivation effects (which could incorporate perceptual, semantic, and emotional attributes of the respective contexts, represented across a wide array of brain regions), it limited our ability to draw conclusions about the role of specific brain structures in supporting context reinstatement and vocabulary recall. Furthermore, as the context-dependent learning enhancement effect was contingent on participants’ subjective sense of presence, future research using newer, more immersive HMD-based VR systems—especially those using omnidirectional treadmills for navigation—may find even stronger context-dependent effects due to the likely increased sense of presence. Additional studies with larger sample sizes will be necessary to characterise more fully how individual differences in presence levels impact the degree of context-dependence in VR learning tasks. Finally, along with CAMIL^40^, recent work has shown that the relevance of an environmental context to the information being learnt in that context is consequential for that information’s memorability^17^ and transfer^41,42^. In our task, the relationship of the contexts to the languages and vocabulary being learnt was completely arbitrary. Future studies may confer additional memory advantages if language learning occurs in VR-based replicas of familiar real-world environments where that language would actually be useful (e.g., learning fruit vocabulary while navigating through the produce section of a grocery store or outdoor farmer’s market). Moreover, investigators should systematically quantify potentially relevant factors such as engagement, intrinsic motivation, interest, and agency in addition to measuring presence.

In summary, this study successfully harnesses context-dependence to enhance the learning of highly challenging and interference-prone material, while remedying the negative effects of context-dependence. After leveraging “contextual crutch” and “desirable difficulties” to enable a rapid learning rate, contextual support and mental reinstatement enabled transfer and overcame context change-induced forgetting, facilitating the real-world retrieval of information learnt in VR. This approach led to strikingly high one-week retention (92%) in participants who received unique contextual support for each language they had learnt, as long as they subjectively perceived the VR-based contexts as actual environments they had inhabited. Moreover, using neuroimaging to quantify mental context reinstatement during vocabulary recall, we found that trials with higher fidelity reinstatement of the learning context were associated a better ability to recall the foreign words they had learnt in that context. As learning and memory are involved in nearly every aspect of life—and they must always occur in some form of contexts—harnessing context-dependence to enhance memory bears far ranging practical implications for education, skill training, health care, as well as a potential to enhance therapeutic learning in evidence-based psychotherapy.

## Methods

### Participants

Data from forty-eight adult participants (26 females, age range 18–27 years; Supplementary Table 1) were included in the analyses for the behavioural experiment; participants were randomly assigned to one of two context conditions (single- and dual-context, each *n* = 24). Data from twenty-two different adult participants (12 females, age range 19–25 years) were included in the analyses for the fMRI experiment; all were assigned to the dual-context condition.

Participants were recruited through flyers posted around the campus of the University of California, Los Angeles (UCLA) and social media advertisements targeting the same geographical area. Participants were tested individually, and they received course credit or were compensated monetarily ($20 per hour for fMRI procedures, $10 per hour for non-fMRI procedures). All participants provided written informed consent, and all study procedures were approved by the Institutional Review Board at the UCLA.

Eligibility screening was conducted using the Research Electronic Data Capture (REDCap) online survey system^43^. Inclusion criteria were as follows: (1) being monolingual English speakers (with no more than high school language courses for any other language) for the behavioural experiment, and being bilingual English speakers (having more than high school language courses for exactly one other language) for the fMRI experiment—this criterion was established for the fMRI experiment to increase baseline recall levels based on pilot results showing that bilingual participants learnt novel foreign vocabulary more quickly; (2) having limited (<5 h) prior exposure to the VR platform used in the experiment; (3) having normal or corrected-to-normal vision and audition; (4) having no diagnosis of learning disabilities; (5) reporting no substance dependence; and (6) not taking any psychotropic medications. Behavioural experiment data from an additional 13 people were acquired but excluded from analyses: five did not complete the procedure due to technical difficulties, three withdrew due to motion sickness during their desktop-VR experience, three did not return for Day 2 procedures, and two were excluded for not following instructions. fMRI experiment data from one additional person was acquired but excluded from analyses, for this individual reported falling asleep during procedure.

### Overview

In the behavioural experiment, participants were randomly assigned to one of the two conditions (single- or dual-context); all participants in the fMRI experiment were assigned to the dual-context condition. All participants underwent the same procedural sequence (Fig. 1): Context A encoding, Language 1 encoding in Context A, Context B encoding, Language 2 encoding in Context A (single-context condition) or Context B (dual-context condition), non-VR test (in laboratory or in MRI scanner), and surprise telephone test.

This experiment measured recall at five time-points (Times 1–5, hence T1–T5). Each language was encoded four times in the VR-based learning contexts: one initial study session followed by three test-study cycles (T1–T3) across two lab visits on consecutive days. At the end of the Day 2 visit, participants were tested outside of the VR learning contexts (T4), either in the lab or in the MRI scanner, and tested again over the telephone one week later (T5).

### Virtual reality

Two distinctive VR-based contexts were used for the learning task (Fig. 2a–d). Participants navigated the world using a computer mouse and keyboard, where the mouse aimed the avatar and the arrow-key press translated to movement in the direction of the given key. They were instructed that the up-arrow (forward motion) was the least likely to lead to simulator sickness. Participants interacted with 3D objects via mouse clicks, and used headphones with a built-in microphone to hear the stimuli and communicate with experimenters. All graphics were displayed on a 27” LED monitor.

“Fairyland Garden” was a fantasy-fiction type context that was bright, verdant, visually open, and expansive. This context’s landscape was rich with water and trees, the buildings were wooden, every room was opened to the outdoors, with birdsongs, crickets, and nature-based ambient sounds (Fig. 2a). “Moon Base,” on the other hand, was a science-fiction type context in which participants were confined indoors within the base, whose structure featured metallic walls, narrow hallways, electronic control panels, artificial colours, mechanical ambient sounds, and participants were always confined indoors (Fig. 2c). Each context contained nine named areas (hence, “rooms”); the names of each room were displayed in English on signs at the boundaries.

The VR-based contexts displayed different experimental objects during the context encoding phase and language encoding phase. During context encoding, location markers were placed in each room to demarcate the location for participants to “stand” as they encoded the context. During language encoding, interactive 3-D objects representative of the to-be-learnt words were placed on “pedestals” in each room, organised along a hinted floor path that displayed transient markers between pedestals (Fig. 2b, d).

An additional VR environment (Fig. 1a.1, 1a.2) was used for participants to learn to control their avatars, receive task instructions, and practice the Context Encoding Task and the Language Encoding Task. This training environment was underwater in honour of one of the pioneering demonstrations of context-dependent memory^4^. It was designed to be visually attractive and highly fantastical (e.g., swimming fishes, shifting lights), so as to allow participants time to adjust to the other-worldly nature of VR experience. This aimed to allow participants to focus on the learning tasks without being distracted by the novelty of the VR experience itself.

These desktop-VR-based contexts were created for this study using the open source OpenSimulator platform (v0.8.2.1, Diva Distribution). Firestorm Viewer v4.4.2-v5.0.7 (2014–2017) rendered content, presented on a computer running Windows 7 Professional. A high-resolution (2560 x 1440) flatscreen display, which participants viewed in close proximity in a darkened room, was used instead of a head-mounted display (HMD). Our initial piloting with an HMD (Oculus RIFT DK1) found that many participants experienced eventual motion sickness that interfered with their ability to concentrate on the task. Switching to an LED monitor (often referred to as “desktop VR”) largely ameliorated this issue, although this may have led to some of our participants reporting a limited sense of “presence” in the VR worlds.

During the VR tasks, an experimenter was present to monitor the behaviour of the participant and to communicate with the participant over headphones. While experimenter and participant were in same room, they were separated by cubicle wall such that they were out of sight from one another.

### Word list, cues, and testing

#### Word list

The to-be-learnt word lists were designed to be as similar, and thus as confusable, as possible. A total of 60 English words, and their translations in two phonetically similar Bantu languages—Swahili and Chinyanja—were used in the experiment. Each participant learnt to pronounce altogether 80 foreign words: 10 learnt in Swahili only, 10 in Chinyanja only, 30 in both languages. The Swahili word list was drawn from Carpenter & Olson (2012)^27^, and the Chinyanja versions of these words were translated using Google Translate™ and modified (see Appendix I. for the word lists and details regarding the modifications).

#### Audio stimuli for language learning and testing

During language encoding, audio recordings of the foreign words accompanied their written form. These recordings were pronounced by a single speaker who had no formal training with Bantu languages (J.K.-Y.E.). This was an intentional decision to ensure the foreign words were readily pronounceable by English speakers, as this experiment prioritised the memory aspect of the task over the degree of linguistic authenticity.

As Smith, Glenberg, and Bjork (1978)^5^ found that experimenters constituted part of the learning contexts, we took precautions to prevent uncontrolled context reinstatement by virtue of subject-experimenter interactions. First, a single speaker recorded audio for both languages during the learning task—to ensure that speaker identity or voice would not serve as context cues between the languages. Every attempt was made by this speaker to not speak to participants during experimental procedures—only providing supervision for the study team in a separate office during the behavioural experiment procedure, and in the fMRI experiment, greeting participants by gestures, then managing equipment in the MRI control room (when asked, participant-facing researchers explained that this person was not to speak to them for scientific reasons, and that the team can answer questions on this matter at the end of their participation). Second, tests that were conducted outside of the learning contexts were cued by other speakers. The English audio cues used in T4 were recorded by A.O., and T5 was conducted by a team of research assistants.

#### Testing software

The short-delay non-VR test (T4; Fig. 4) was presented using PsychoPy2^44,45^. The long-delay surprise memory test was administered over telephone calls using Google’s Hangouts™ communication platform (audio-only), digitally recorded with participant permission, with foreign vocabulary recall cued conversationally by experimenters.

### fMRI protocol and in-scanner verbal response recording

#### fMRI protocol

fMRI data were collected with a Siemens 3.0 Tesla Magnetom Prisma scanner at the UCLA Ahmanson-Lovelace Brain Mapping Center, using a 64-channel head coil. Functional data were acquired using T_2_*-weighted simultaneous multislice echoplanar imaging (EPI) sequences (TR = 1.0 s; TE = 30 ms; flip angle = 52°; FoV = 20.8 cm; multiband acceleration factor = 5; 65 oblique axial slices; voxel resolution 2 × 2 × 2 mm). Each of the 10 runs consisted of 330 volumes and included eight trials of the task (we did not discard initial volumes as the version of Syngo software did not begin recording until T1 stabilised). Additionally, a T1-weighted structural MRI [axial magnetisation-prepared rapid gradient-echo (MPRAGE), 0.8 mm^3^] was obtained for spatial registration of the functional data.

Auditory stimuli were presented via OptoActive™ noise cancelling headphones, which were equipped with the FOMRI III™ + microphone (Fig. 1c) to record participants’ verbal responses during fMRI scans. This system provided online noise cancellation, which enabled high-quality recordings of participants’ vocalisations and allowed participants to clearly hear the audio stimuli despite the scanner noise. No post-experimental denoising of the verbal response was required. Button responses were recorded via CurrentDesign Fibre Optic Response Pads, an MR-compatible button box device. MR-compatible goggles were used to for visual presentations.

### Procedure: day 1 and day 2, context and language encoding (T1–T3)

#### Day 1

##### Familiarisation, instructions, and practice

After informed consent and general instructions, participants “entered” the introductory VR environment. Therein, participants first familiarised themselves with the navigational controls. They then received instructions for the context- and language encoding tasks by watching a video on a screen within the world (Fig. 1a.1, Supplementary Video 2), and practiced the two tasks (Fig. 1a.2) under the supervision of an experimenter, who provided corrective feedback to ensure that participants had proper understanding of the tasks. Participants practiced the context encoding task (see below) by performing it in the practice context. Then they practiced the language encoding task by learning the translations of a set of practice items in the pseudo-language ‘Pig Latin’.

##### Context A encoding (Fig. 1a.3)

Participants were then “teleported” to Context A (Moon Base or Fairyland Garden, counterbalanced across participants), where they performed a guided encoding task of the VR-based context itself. Each context contained 9 “rooms,” each equipped with a location marker. In each room, participants were instructed to walk to the marker and do two full clock-wise rotations (720°) within 30 s while looking around the room. Participants were instructed to pretend that they were a tourist who had forgotten their camera and that they should try to remember what it felt like to be in that particular place. As participants entered and exited each room, the experimenter informed participants the names of the rooms (e.g., “You are now leaving Sickbay and entering Airlock.”).

##### Language 1 encoding (T1–T2; Fig. 1a.4, Supplementary Video 1)

There were four rounds of language encoding for each language (three rounds on Day 1, and one on Day 2). Before each round, participants were told which language they would be learning. After Context A encoding and a mandatory 2-min break, participants re-entered Context A for Round 1 of Language 1 encoding (Swahili or Chinyanja, counterbalanced across participants).

In each round, participants navigated along the hinted walking path (Fig. 2b, d) and encountered a series of 40 pedestals (with 3–5 pedestals in each room). Upon each pedestal hovered a slowly rotating, 3-D object representation of the to-be-learnt word (e.g., a rooster), with its English name floating above to ensure that participants could have certainty about what that object was (i.e., so they knew it was not a hen or turkey). As Fig. 2e denotes, participants were instructed to walk up to each object, read its English name aloud, and then to click on it. The click changed the floating English text to reveal the foreign transliteration, and participants would hear the foreign pronunciation three times via headphones, evenly spaced across 10 s. Participants were instructed to repeat after the audio each time by pronouncing the foreign word aloud. Upon completion, they would then click the pedestal to reveal a visible path marking the way to the next pedestal with the next object. The path hints were transient and disappeared after use. Object sequences were controlled so that they were consistent within each language. That is, for a given participant, the same object always appeared in the same location for one language, but always in a different location for the other language. The pedestal locations and navigational route remained consistent across all rounds. A 5-min break was inserted between Rounds 2 and 3.

##### Retrieval practice (Fig. 2e.2)

Retrieval practice was incorporated into Rounds 2–4. During Rounds 2–4, after participants walked up to each object and spoke aloud its English name, they were to first attempt to verbally recall its foreign translation before clicking the object. If the participant did not recall the translation and did not wish to attempt a guess, they had the option to say “pass.” They then clicked the object, which triggered the transliteration of the foreign word to the appear and the audio of its pronunciation to be played. Thus, regardless of whether they were correct, incorrect, or passed, the participant received feedback as to the correct answer. Then, as with Round 1, participants heard and repeated after the audio three times within a 10 s period. Participants’ verbal responses were digitally recorded and used to index their memory recall ability during each round, with performance summarised as: T1 (recall during Round 2 before the 2nd encoding), T2 (recall during Round 3 before the 3rd encoding), and T3 (recall after an overnight delay, before the 4th and the final encoding). In the rare cases when participants neglected to attempt recall or say “pass” before clicking an object, the associated vocabulary words were dropped from analysis after that time point. For example, consider a participant who clicked the 3-D boat object during Round 3 before attempting to recall the Swahili word for “boat.” Even though the participant would continue to encounter the boat in Round 4 to maintain consistency across participants, that word would be excluded in analyses of that participant’s T3, T4, and T5 data.

##### Context B encoding (Fig. 1a.5)

After Round 3 of Language 1 encoding, participants encoded Context B. The procedure was identical to Context A encoding, except it occurred in the other VR-based context. This was followed by a 5-min break.

##### Language 2 encoding (T1–T2; Fig. 1a.6)

After the break, participants began Language 2 encoding. This is the only portion of the procedures in which the experiences of the two context groups diverged. Dual-context participants remained in Context B to encode Language 2, while single-context participants were teleported back to Context A to encode Language 2 (note that single-context participants never learnt any language in Context B). The encoding procedure was identical to Language 1 encoding.

##### Post-VR questionnaires

Thereafter, participants completed on REDCap^43^ a presence scale used in a prior study^19^, an immersion survey (this survey was not used in the analysis)^18,46^, the Simulator Sickness Questionnaire^47^, and the Pittsburgh Sleep Quality Index^48^. They were then reminded of their appointment the next day, and sent home.

#### Day 2

Participants returned the next day around the same time of day to perform *Language 1 Encoding Round 4* (T3). Then, following a 2-min break, participants performed *Language 2 Encoding Round 4* (T3). Round 4 was participants’ last exposure to the foreign words and VR contexts.

### Procedure: day 2, short-delay, non-VR testing (T4)

Language encoding was followed by a 10-min break (behavioural experiment) or 30-min break (fMRI experiment), after which participants were tested for the first time outside of the VR-based learning contexts (T4), either in the lab (behavioural experiment) or in the MRI scanner (fMRI experiment). During the break, participants in the behavioural experiment were unoccupied for 10 min under supervision, seated in a waiting room without using internet-capable devices. A 30-min interval was scheduled for participants in the fMRI experiment. During this time, each participant was escorted by their experimenter to the Ahmanson-Lovelace Brain Mapping Center (an 8-min walk from the laboratory), underwent final MRI safety screening, and was set up in the MRI scanner.

T4 consisted of 80 trials (one for each foreign word learnt) evenly divided into 10 runs. Each trial (Fig. 4) consisted of the following periods: “Ready” screen, mental reinstatement, language recall, imagery vividness rating, and two trials of an arithmetic task that served as active baseline for fMRI data analysis. T4 procedures were identical in the behavioural and fMRI experiments.

#### Ready (1 s)

A grey screen with the words “Get Ready” printed was presented to mark the beginning of each trial.

#### Mental reinstatement (10 s)

The mental reinstatement period began with an audio cue for each trial, which stated the name of a VR-based context, followed by that of a room therein (e.g., “Moon Base: Airlock”). Following the audio cue, the screen turned black, and based on instructions provided to the participants before the scan, they knew that this meant that they should close their eyes, imagine themselves back in that specific room, and mentally perform the full rotations (as they had practiced the prior day in the VR-based context encoding task) until they heard a beep. Participant used a series of button presses to indicate the progress of their imagined rotation: mentally “placed” themselves on the marker, rotated 180°, 360°, 540° and so on. If participants completed a full rotation before the allotted time, they were instructed to continue mentally rotating and button-pushing until the beep. Upon hearing the beep, which sounded 10 s after audio cue offset, participants were to cease performing the mental rotation task and open their eyes to prepare for the next phase of the trial.

In the *congruent* reinstatement condition, participants were cued to reinstate the specific room in which they had learnt the word to be recalled later in this trial. In the *incongruent* condition, they were cued to reinstate a room from the other context (for dual-context participants, this was the context where they had learnt the other language; for single-context participants, this was the context where they had not encoded any language). These conditions were pseudo-randomly intermixed.

#### Language recall (8 s)

The language recall period began 2 s after the onset of the previous beep. Participants first heard an audio cue, which stated a language, then an English word whose translation they had learnt in the stated language (e.g., “Chinyanja: rooster”). After hearing the cue, participants were to covertly retrieve the English word’s translation in the cued language (i.e., to mentally recall the foreign word without saying it aloud). If they felt they were successful, they were to push Button 1 and to continue thinking about the word until they heard a beep. If they failed to retrieve the foreign word, they were to push Button 2 and continue to attempt retrieval until the beep—should they succeed at any point after indicating failure, they were to push Button 1 at the moment of successful retrieval. The beep sounded 8 s after the cue offset, at which point participants were to verbally pronounce the foreign word, or as much of it as they could remember. These responses were recorded and scored as T4 data. The length of the verbal response recording period varied between 6.5–7.0 s depending on the length of the cue (3.0–3.5 s), so that the combined duration of the two always summed to 10 s.

#### Imagery vividness rating (2 s)

After verbal recall, participants were then asked to rate how vivid the previous mental reinstatement had been (1 for very vivid, 2 for vivid, 3 for not vivid, and 4 for unsuccessful). These ratings were later used for trial exclusion during the analyses involving mental reinstatement.

#### Arithmetic task (5 s)

At the end of each trial, participants performed an arithmetic task. Participants saw a display (2.5 s) with two single-digit integers, and they were to push Button 1 if the product of these numbers was odd, and Button 2 if even. Then a new pair of digits appeared (2.5 s) and participants performed the same task.

### Procedure: day 2, post-experimental survey

After T4, participants completed a short survey to ask them about what strategies (if any) they had implemented to learn and recall the words, and if there was anything else they would like to communicate to the experimenters.

### Procedure: day 8, one-week delay, surprise testing (T5)

On Day 8, participants were telephoned for a scheduled “follow-up interview” with the understanding that an experimenter would “ask them about things they had experienced in the VR.” The only instruction they received about the phone call was that they were to be at home, seated in a quiet place. Participants were not informed that they would be tested again.

During the call, the experimenter requested permission to record the participant’s responses. After permission was granted, experimenter asked the following questions: (1) Had they looked up or studied any of the Swahili or Chinyanja words during the preceding week? (2) Had they expected to be tested again? (3) What percentage of the words did they expect to recall? (see Supplementary Note 3).

The experimenter then conducted a cued recall test to test participants’ memory for all 80 of the foreign words they had learnt. On each trial, the experimenter cued the participant with an English word and a language that it was to be translated into (e.g., “How do you say ‘cherry’ in Swahili?”). The order in which the words are tested was fully randomised, such that testing hopped back and forth between the two foreign languages. Participants’ vocal responses were recorded and scored as T5 data.

### Language test scoring

#### Recall

Digital recordings of the verbal responses from T1–T5 were scored offline by two scorers. The score for each word was the number of correct phonemes divided by the number of total phonemes. Scorers were trained to use a detailed decision tree, and when the two scorers disagreed, the average between the two scores was used as the final recall score for that word. The partial word score was used to provide more fine-grained results than binary (correct *vs* incorrect) word recall. In this scoring scheme, phonemes in shorter words were weighed more heavily than phonemes in longer words. This weighting mirrors the consequences of phonemic errors in real-world communication. When one mistakenly places, for instance, a “P” instead of an “V” in the word “van” it tends to be more consequential than in a longer word like “supervisor,” and a lot more difficult for the listeners to guess the intended meaning.

#### Retention measures

Retention was measured inversely via a forgetting score between two tests. Overnight retention (reported in Supplementary Note 1: A4) was computed based on the difference between T3 and T2. One-week retention was computed based on the difference between T5 and T4.

##### Forgetting score

The forgetting score was computed as follows: First, an item-wise forgetting index was computed for each word with a non-zero score in the earlier test (i.e., if no phonemes were recalled in T4, the word was excluded from this computation for one-week forgetting). These forgetting indices measured loss between the two tests: a negative forgetting index would mean the word was recalled worse after one-week, and a forgetting index of zero would mean no forgetting, thus perfect one-week retention. For example, consider a word that had a recall score of 1 (full, correct recall) on T4, but only 0.5 (half of the phonemes were missing or incorrect) in T5. It would receive a “−0.5” on the forgetting index, indicating half of the word had been forgotten. On the other hand, if a word had a score of 1 on both T4 and T5, it would receive a “0” on the forgetting index, indicating perfect retention. These forgetting indices were then averaged within each participant (across all eligible words) to produce a forgetting score. The forgetting score was a metric of forgetting, or the inverse of retention—the more negative the score, the more forgetting and thus the poorer retention.

##### Retention score

For the ease of interpretation, a positive retention score was computed by 1 minus averaged forgetting score. In which 1 indicates perfect retention across all eligible words, 0.5 indicates half of the information was retained, while 0 means no information were retained.

#### Intrusion measure

When scoring T4 and T5, scorers were instructed to compare the transliteration of each word to its counterpart in the other language, and to determine from experience whether the word in question was similar to any other words in either language (see Appendix II for intrusion coding). The scorers were experimenters who became highly familiar with the words in both languages. In addition to formal training, scorers spent 2–6 h each week monitoring participants during language encoding, testing participants during T5, or scoring verbal response offline. Despite this, “similarity” between words remains arbitrary and experience-based. Therefore, two cautions were introduced: a newer scorer was always paired with a very experienced one in the scoring assignments, and the maximum code was used when the scorers disagreed—as the higher ratings denote more severe intrusions, and preliminary examination revealed that novice scorers tend to underrate intrusion rather than overrate them.

### Behavioural data analysis

Multiple statistical tests were conducted using SPSS 26.0^49^. The between-subject factors were Context Group (single- *vs*. dual-context) and Presence (high- *vs*. low-presence, a mean-split grouping using the presence scale^19^). The within-subject factors were Times (T1–T5), Language Order (Language 1 *vs* 2; not reported, see Supplementary Note 1: A1), and Reinstatement (congruent *vs*. incongruent reinstatement). The dependent variables were intrusions (number of items coded to be intrusions from the opposite language, out of a total of 80 items), recall (mean of item-wise percentage phonemes correct for a given test), and retention (see *Retention Score* above).

### fMRI data analysis

#### fMRI pre-processing

Functional data were pre-processed without spatial smoothing, pre-whitening, nor B0 unwarping using the FMRI Software Library 5.0.4 and Advanced Normalisation Tools (ANTS 2.0)^50^. FSL Brain Extraction Tool (BET2)^51^ was used to perform brain extraction. FSL^52^ FEAT^53^ was used to apply a high-pass temporal filter (128 Hz). Timeseries alignment, motion correction, and registration to standard Montreal Neurological Institute (MNI) template was performed using FMRIB’s Linear Image Registration Tool (FLIRT)^54–56^, Motion Correction FLIRT (MCFLIRT)^54^, and ANTS.

#### fMRI task timing and trial categorisation

The mental reinstatement (Fig. 4 “Imagery”) and language retrieval (Fig. 4 “Language”) periods from each trial were extracted from the dataset. The BOLD timeseries for these periods were extracted using the adjusted onset and offset times (5 s, i.e., 5 TRs, were added to onsets and offsets to account for the lagging hemodynamic response, or HDR). The resulting truncated timeseries was then temporally averaged at each voxel, yielding one averaged imagery pattern and one averaged language pattern for each trial.

##### Imagery

Each “Imagery” period began when participants indicated that they had mentally “placed” themselves in the to-be-reinstated context via a button push (Fig. 4 “Orient”), and end at the beep onset (the beep which informed participants to open their eyes and end mental reinstatement). The onset for each trial was based on participants’ responses, thus the imagery period duration varied in length. Imagery period data were labelled as Moon base or Fairyland Garden, based on the world that participants were cued to reinstate. Trials were excluded if participants reported they were “unsuccessful” during the imagery rating portion, or did not push buttons to report mental reinstatement rotation progress.

##### Language

Each “Language” period began with the onset of the audio cue, and ended 6 s afterwards. The duration of this period was task-based, and fixed in length. Language period data were labelled by the foreign word to be recalled (e.g., Chinyanja: Dress).

#### Searchlight multi-voxel pattern analysis (SL-MVPA)

A SL-MVPA was conducted using the Imagery patterns to identify regions in the brain that expressed multivariate patterns of activity capable of discriminating between a participant’s mental reinstatement of Moon Base vs. Fairyland Garden (Supplementary Fig. 1). To this end, we employed a support vector machine (SVM) classifier with a linear kernel using libSVM (c-SVC, c = 1)^57^ and a whole-brain searchlight mapping approach (radius = 4 voxels). Classification was cross-validated using a leave-one-run-out method—the classifier was trained on valid trials from 9 runs (9 × 8 trials), and tested on the valid trials from the left-out run (8 trials). Trial labels were balanced prior to classification by randomly sampling from the overrepresented trials to match the underrepresented trial types. The entire cross-validation procedure was repeated over 10 iterations (one for each run) and the classification results were averaged. This produced a brain map whose voxel values reflected the classifier’s cross-validation accuracy when the searchlight sphere was centred on that voxel (Supplementary Fig. 1.4). The top 2000 voxels with the highest classification accuracies were identified for each participant, and used to create a distributed region of interest for the subsequent representational similarity analysis as a within-subject feature selection (Supplementary Fig. 1.5).

#### Representational similarity analysis (RSA)

For each word that each participant had learnt, the RSA produced a value of similarity between (1) the brain response pattern when the participant was recalling this word, and (2) the averaged brain response pattern when the participant was mentally reinstating that word’s learning context (Fig. 5a).

This within-subject RSA was conducted using custom MATLAB code. First, trial-specific imagery and language patterns (produced by the aforementioned temporal average of HDR-adjusted timeseries within trial period) for each participant were masked using the participant’s top 2000 voxels identified in the SL-MVPA. Second, the imagery patterns for each learning context were averaged within-subject to produce a participant-specific mental reinstatement template for Moon Base and Fairyland Garden. Third, the language pattern for each word was then correlated (Pearson’s *r*) with the reinstatement template of its learning context. For instance, consider a participant who had learnt “banana” in Chinyanja in Fairyland Garden. The language period during the covert retrieval of the word “banana” in Chinyanja would be correlated with the Fairyland Garden template—an average of all imagery patterns during the mental reinstatement of Fairyland Garden. Fourth, the resultant *r*-values were Fisher transformed to normally distributed *z*-values to allow for comparison across trial-types. Lastly, a mean split was performed on the *z*-values to categorise each trial as either a high-fidelity reinstatement trial or a low-fidelity reinstatement trial to analyse the verbal response data.

#### Repeated measure analysis of variance (RM-ANOVA)

A 2 × 2 × 2 × 2 RM-MANOVA was performed on with the factors Times (T4, T5) × Reinstatement instructions (congruent vs incongruent) × RSA (high- vs low-RSA) × Presence (high- vs low-presence) on recall using SPSS 26.0^49^. The dependent variables were proportion syllables recalled during T4 (short-delay recall in the MRI scanner) and T5 (one-week-delayed recall over the telephone).

### Reporting summary

Further information on research design is available in the Nature Research Reporting Summary linked to this article.

## Supplementary information

Demonstration of Language Encoding Task

Day 1 Instructional Video for Participant

Revised Supplemental Material

Reporting Summary Checklist

Supplementary Videos Legends

## Data Availability

De-identified data available from the corresponding author, upon request.

## Code availability

The MATLAB scripts used for fMRI data preprocessing and statistical analysis are available from the corresponding author, upon request.
