# Supplementary material for: Enhancing learning and retention with distinctive virtual reality environments and mental context reinstatement
Source: NPJ Sci Learn. 2022 Dec 8;7:31. doi: 10.1038/s41539-022-00147-6 (PMC9732332; doi:10.1038/s41539-022-00147-6)
Supplement: Supplementary file 3 — Revised Supplemental Material [file 41539_2022_147_MOESM3_ESM.docx]

**Supplementary Materials**

**Supplementary Table 1 | Demographic information of participants.** The mean (SD) ages of participants.

|  | Male | | Female | | Overall | |
| --- | --- | --- | --- | --- | --- | --- |
| Behavioural Exp. Sample | n | Age | n | Age | n | Age |
| Single-Context | **11** | **20.7 (1.1)** | **13** | **19.5 (1.1)** | **24** | **20.0 (1.2)** |
| *High Presence* | 5 | 20.6 (1.1) | 8 | 19.6 (1.1) | 13 | 20.0 (1.2) |
| *Low Presence* | 6 | 20.8 (1.2) | 5 | 19.2 (1.1) | 11 | 20.1 (1.4) |
| Dual-Context | **11** | **20.9 (2.5)** | **13** | **18.7 (0.9)** | **24** | **19.7 (2.1)** |
| *High Presence* | 6 | 21.5 (3.0) | 4 | 19.0 (1.4) | 10 | 20.5 (2.7) |
| *Low Presence* | 4 | 20.5 (1.9) | 9 | 18.6 (0.7) | 13 | 19.2 (1.5) |
| *Missing Presence Data* | 1 | 19 | 0 | N/A | 1 | 19 |
| Overall | **22** | **20.8 (1.9)** | **26** | **19.1 (1.1)** | **48** | **19.9 (1.7)** |
| fMRI Exp. Sample | n | Age | n | Age | n | Age |
| *High Presence* | 3 | 20.0 (1.7) | 9 | 21.8 (1.7) | 12 | 21.3 (1.8) |
| *Low Presence* | 6 | 20.8 (1.3) | 3 | 19.7 (1.2) | 9 | 20.4 (1.3) |
| *Missing Presence Data* | 1 | 21 | 0 | N/A | 1 | 21 |
| Overall | **10** | **20.6 (1.4)** | **12** | **21.2 (1.8)** | **22** | **20.9 (1.6)** |

**Supplementary Table 2 | Presence scores.** The mean (SD) presence scores of participants from a 10-item presence scale (range = 1-5).

|  | *Low Presence* | | *High Presence* | | Overall | |
| --- | --- | --- | --- | --- | --- | --- |
| Behavioural Exp. Sample | n | Pres. Score | n | Pres. Score | n | Pres. Score |
| *Single-Context* | 11 | 1.7 (0.4) | 13 | 3.4 (0.5) | 24 | 2.7 (1) |
| *Dual-Context** | 13 | 1.9 (0.3) | 10 | 3.2 (0.4) | 24 | 2.5 (0.7) |
| Overall | **24** | 1.8 (0.4) | **23** | 3.3 (0.5) | **48** | 2.6 (0.9) |
| fMRI Exp. Sample | n | Pres. Score | n | Pres. Score | n | Pres. Score |
| *Dual-Context** | 9 | 1.9 (0.6) | 13 | 3.4 (0.5) | 22 | 2.8 (0.9) |

Each * Denote a group that contained one participant with missing presence score.

Supplementary Figure 1. | fMRI experiment: MVPA-based feature selection.

**
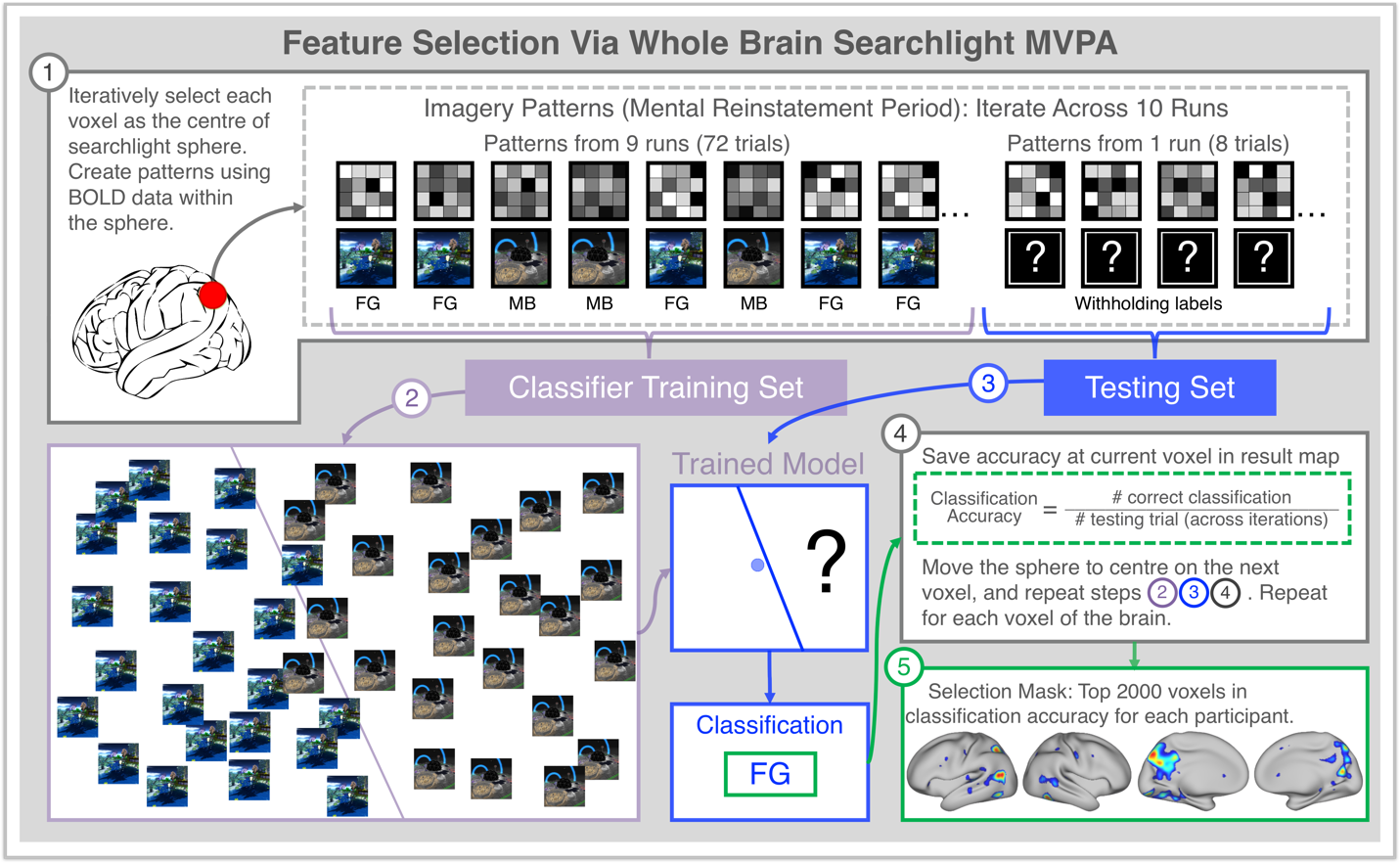
Supplementary Figure 1 | fMRI experiment: MVPA-based feature selection.** After pre-processing, imagery and language patterns for each trial were created using fMRI data from the mental reinstatement and word recall periods (see Figure 2). Feature selection: a within-subject, whole brain searchlight multivoxel pattern analysis (SL-MVPA) was conducted using imagery patterns, which identified and mapped the 2000 voxels containing the most discriminative information (i.e., highest classification accuracy values) to classify between the mental reinstatement of Moon Base vs. Fairyland Garden for each participant. **1**, A searchlight sphere with a 4-voxel radius was iteratively centred on each voxel in the brain, and the imagery pattern data within the voxels residing within each sphere were used for MVPA classification. The classifier was trained on 9 out of 10 runs of data (72 trials) to discriminate between Moon Base (MB) and Fairyland Garden (FG). **2**, For each sphere, the support vector machine (SVM) classifier determined a hyperplane that maximally discriminated between the classes within the training set. **3**, The trained classifier model then made a classification for each trial of the testing set (patterns from each of the 8 trials in the held-out run, with their labels withheld), determining whether the trial was a MB or FG mental reinstatement. **4**, After Steps 2-3 were repeated for each of the 10 runs (i.e., each run had served its turn as the testing set), the classification accuracy for the sphere was computed (correctly labelled trials ÷ total number of trials) and saved in the SL-MVPA results map, at the location of the present voxel. **5**, Accuracy values within the SL-MVPA map were ranked and voxel locations containing the 2000 highest classification accuracies were used as a distributed mask for the participant. All patterns in the subsequent analytic steps were masked with these voxels (i.e., all data outside of the mask were excluded from analyses).

**Image attribution:** This figure was modified from one J.K.-Y.E. had created for a book chapter she previously published with J.R. in *the Handbook of Research Methods in Human Memory.* The VR imaged here were created by J.K.-Y.E. using models created by her or by Mr. Forde Davidson (“JubJub Forder”) as commissioned by the research team, or were from the OpenSim community shared under the Creative Common 0 License (public domain). The icons used were created by J.K.-Y.E. or were modified from stock icons in MS PowerPoint or public domain.

Supplementary Figure 2. | Group mean searchlight decoding map.


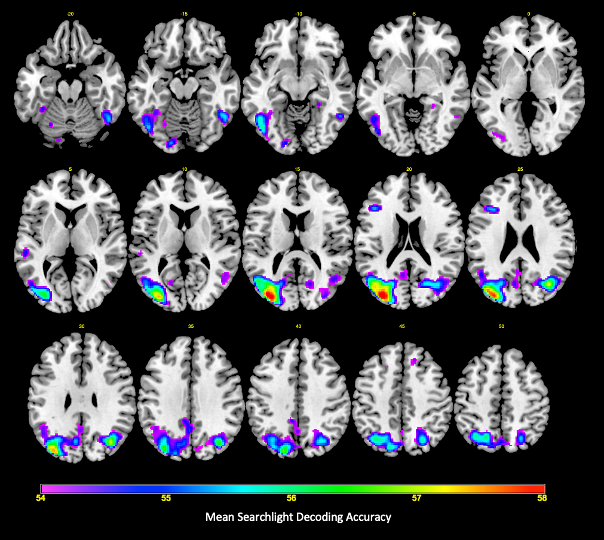


Supplementary Figure 2. | Group mean searchlight decoding map. Whole-brain searchlight accuracy maps of all fMRI participants were averaged to create a group map illustrating the regions that showed the strongest ability to decode whether participants were imagining themselves at a location in the Moon Base or Fairyland Garden world during the mental reinstatement phase of individual trials. Peak decoding was observed in bilateral visual association regions (superior lateral occipital cortex, ventral occipito-temporal cortex, fusiform gyrus, medial parietal regions (precuneus, posterior cingulate cortex), lateral parietal regions (intraparietal sulcus and superior parietal lobule), and the left inferior frontal sulcus. Given that all of our fMRI analyses were conducted within-subject (i.e. selecting the top 2000 voxels from each participant’s searchlight map and creating RSA templates using those voxels), this group map is presented for illustrative purposes only and arbitrarily thresholded.

A. Supplementary Note 1 (Results of the Behavioural Experiment)

***A1. T1-T3 Recall secondary results (confirming controls and design)***

A repeated measure analysis of variance (RM-ANOVA) was performed on T1-T3 recall examining the effects of Times × Language Order × Context Group × Presence. Language Order was entered as a factor to confirm that the context groups did not differ in Language 1 learning at T1 or T2 (*p*>.05), as the experience of the groups would have been identical up to that point. A significant interaction of Times × Language Order was found (*F*(1.6,1.8)=7.41, *p*<0.01, *η_p_*^2^= .18). Language 2 was better recalled than Language 1 only at T1 and T2 (*p*<.05), and performance on the two languages converged after an overnight delay at T3 (*p*>.05).

***A2. Mental Reinstatement on T4 Recall***

A 2 × 2 × 2 multivariate ANOVA (MANOVA) was performed with the factors Reinstatement × Context Group × Presence on T4 performance (n=42). Six participants had insufficient trial counts for successful mental reinstatement; thus, entering mental reinstatement as a factor into the main RM-ANOVA (see Supplementary Data 1: A3) would have led to the exclusion of these 6 participants. Therefore, we conducted this analysis as a separate MANOVA in order to not exclude the 6 participants in the overall analysis. A significant multivariate main effect of reinstatement was found (*F*(4,35)=3.99, *p*=.009, *η_p_^2^*= .31). Follow-up univariate analysis revealed a main effect of reinstatement on recall (*p*=.04). Post hoc contrast with Bonferroni correction showed that recall was higher in the congruent (.52 ± .18) as compared to the incongruent reinstatement condition (.47 ± .19). A similar exploratory analysis was also performed to examine whether reinstatement condition at T4 affected performance on T5, where no significant effect was found.

***A3. Times, Context Group, and Presence on T4, T5 Recall and Intrusions***

A 2 × 2 × 2 RM-MANOVA was performed on with the factors Times × Context Group × Presence on T4 and T5 intrusions and recall. A main effect of Context Group was found on intrusions (**Figure 3c**; *F*(1,43)=6.49, *p*=.02, *η_p_^2^*= .13), in which the single-context group had more intrusion items (6.57 ± 4.69, out of 80 total items) than the dual-context group (4.09 ± 4.82). Additionally, a significant interaction of Times × Context Group on recall was found (*F*(1,43)=6.28, *p*=.014, *η_p_^2^*= .13). However, the simple main effect of Context Group on recall measures did not reach significant in the follow-up one-way ANOVAs. Moreover, a significant Times × Presence interaction on recall was found (*F*(1,43)=4.73, *p*=.04, *η_p_^2^*= .10). Follow-up one-way ANOVA (*F*(1,45)=4.81, *p*=.03) revealed that high-presence participants (.39 ± .19) recalled more on T5 than those reporting low presence participants (.28 ± .17; *p*=.04).

***A4. Overnight and One-Week Retention***

A 2 × 2 × 2 RM-MANOVA was performed with the factors Times × Context Group × Presence on overnight and one-week retention. These dependent variables could not be entered as separate measures in the previous analysis because the Times levels involved were different (this analysis examined T3 and T5 retention, whereas the previous analysis examined T4 and T5 recall). A significant multivariate interaction was found between Context Group and Presence across the retention measures (*F*(1,42)=5.22, *p*=.03, *η_p_^2^*= .11). Trends of simple interactions were found for both measures (*p*=.062 for overnight, and *p*=.081 for one-week retention), and post hoc tests with Bonferroni correction were conducted. Amongst low-presence participants, the single-context (.71 ± .13) and dual-context (.75 ± .13) groups did not perform differently in one-week retention (*p*=.47). However, amongst high-presence participants, the dual-context group (.92 ± .07) retained significantly more items than the single-context group (**Figure 3d**; .76 ± .12, *p*=.002). All overnight retention simple contrasts were n.s. (all *p* > .05).

B. Supplementary Note 2 (Results of the fMRI experiment)

The fMRI sample did not have a comparison group, as only within-subject effects were of interest. Therefore, only T4 and T5 data were analysed. We first conducted a 2 × 2 × 2 × 2 RM-MANOVA on the recall data with the factors Times × Reinstatement instructions × RSA × Presence, and one participant was excluded for this analysis due to missing data on self-reported presence. We found no significant effects of presence, and the n.s. between-subject effect showed *η_p_^2^*=0.09. Therefore, we proceeded to exclude presence as a factor in our fMRI analyses, and we retained the fMRI data from the participant who failed to complete the presence survey. The effects that reached significance did not differ across the two models with and without the factor of presence.

***B1. Times, Reinstatement Instructions, and Reinstatement Fidelity on Recall***

A 2 × 2 × 2 RM-MANOVA was performed on with the factors Times × Reinstatement instructions × Reinstatement Fidelity on recall.

A significant main effect of Times was found (*F*(1, 21)=72.27, *p*<.001, *η_p_^2^*=0.78), where T4 recall (.54 ± .17) was greater than T5 recall (.42 ± .17).

A significant main effect of Reinstatement Fidelity was found (**Figure 5b,** *F*(1, 21)=13.712, *p*=.001, *η_p_^2^*=0.395), where recall on high Reinstatement Fidelity (.50 ± .17) trials was better than recall on low Reinstatement Fidelity (.45 ± .18) trials across T4 and T5.

A significant Reinstatement Instructions × Reinstatement Fidelity interaction on recall was found (**Figure 5c, 5d**; *F*(1, 21)=6.59, *p*=.02, *η_p_^2^*=.24). Follow-up simple contrasts with Bonferroni correction revealed that after congruent mental reinstatement, recall did not differ across high Reinstatement Fidelity (.50 ± .19) and low Reinstatement Fidelity (.48 ± .18) levels (*p*=.346); however, after incongruent reinstatement trials with high Reinstatement Fidelity (.51 ± .16) resulted in 8.5% higher recall than low Reinstatement Fidelity trials (.43 ± .18; *p*=.001). Although no significant Times × Reinstatement Instructions × Reinstatement Fidelity interaction was found, it was of theoretical interest to examined if the Reinstatement instructions × Reinstatement Fidelity interaction differed across short- and one-week-delayed recall. Simple interaction revealed that the Reinstatement instructions × Reinstatement Fidelity interaction was driven by one-week-delayed recall (**Figure 5d**; *p*=.006), while short-delay recall showed a trend but did not reach significance (**Figure 5c**; *p*=.33).

C. Supplementary Note 3 (Qualitative Data)

***C1. Telephone Interview***

During the one-week delayed telephone call, the experimenter requested permission to record the participant’s responses and asked a series of questions. (1) Had they looked up or studied any of the Swahili or Chinyanja words during the preceding week? Only one participant out of 71 reported having looked up some of the words. (2) Had they expected to be tested again? Only five out of 71 participants either expected a test, or had suspected the “telephone interview” would be a surprise test. (3) What percentage of the word did they expect to recall? 49 participants expected to recall <20%, only 4 participants expected to recall more than 50%. While there was a correlation between expected recall and actual recall (*r*=.62, *p*<.001), 87.1% of participants (n=61) recalled more than they expected by an average of 21.2% (*SD*=13.4%).

D. Supplementary Discussion

***D1. Further Consideration of the Potential Implications of our Findings***

***Implications for research on learning: cognitive, clinical sciences, and beyond.*** The present findings offer possible avenues to improve experimental control and to expand the experimenters’ toolkit for research that requires participants to learn and recall information. First, many experiments—ranging from clinical studies to cognitive and educational sciences—require people to learn and recall information across contexts. For instance, participants in a clinical trial may learn therapeutic skills in the clinic during psychotherapy, then need to utilise these skills beyond the clinic (e.g., home, school, workplace). Alternatively, in a cognitive psychology study, participants may view stimuli in the laboratory, then be asked to recall or make a decision/ judgment about these stimuli in some other context (e.g., while undergoing neuroimaging procedures).

Let us consider neuroimaging studies as an illustrative example of this issue. The physical context change between non-neuroimaging and neuroimaging procedures can be quite salient. In the former, participants may be seated at a desk before a monitor. In the latter, they may lay supine in a noisy, cold MRI scanner, or wearing EEG caps, imposing a limited range of motion. Unless accounted for, this change of context could lead to change-induced forgetting that may confound the results. As context-dependence based effects grow in magnitude with longer retention intervals, this poses a particular issue for longitudinal studies. Our data show that mentally reinstating the learning context presents a possible solution to bridge the context change to enable the transfer of the previously learnt information.

***Implications for pedagogy: education, military, occupational, and other skill-based training.*** The success in leveraging context-dependence to enhance memory bears importance for pedagogy in its various forms; for example, school- and university-based education, self-guided learning, and skill training such as involved in military and occupational settings. First, trainees and students are often called upon to learn information and skills, then recall it in a different context. These results suggest that mentally reinstating the learning contexts during recall would enhance *transfer* and improve recall performance. For example, after learning Spanish in a quiet classroom, a national guardsperson needs to recall what she learnt while communicating with a Spanish-speaking citizen in a bustling scene of hurricane recovery effort. While the context of recall maybe drastically different from the learning context, mentally placing herself in that classroom would aid her in recalling the information she had learnt.

Second, trainees and students usually are trained in multiple skill sets or subjects within the same time frame. Our findings showed that the combination of distinctive contexts and mental reinstatement facilitated the simultaneous learning of multiple information sets. For instance, consider a scenario where the same national guardsperson learnt a series of Latin-based medical terms in her first-responder training, while she also learnt Spanish. These information sets could interfere with one another and cause confusion during recall. Our results suggest that she can improve recall and reduce interference if she studies the two subjects each in its own distinctive and unique settings, and then mentally reinstates the appropriate study context during recall.

Third, while distinctive contexts can provide unique cues to bind with memory to reduce interference, distinctive contexts could be difficult to identify or time-consuming to travel to. Our results demonstrated that techniques to harness context-dependence can be applied using VR-based contexts, which reproduced the effects of physical contexts. Although our results showed that context effects can be achieved even with ‘desktop VR’, affordable commercial VR headsets enable access to distinctive and highly immersive VR contexts that can be instantaneously traversed. This makes the applications of context-related learning techniques feasible and accessible—especially for trainees and learners with mobility challenges.

Lastly, the potential uses of VR as a pedagogical tool have been increasingly discussed and tested over the past decade. VR allows trainers and educators to offer trainees and students opportunities that are otherwise impossible or dangerous (e.g., visiting historical Roma or the Smithsonian Museum when it is closed due to a pandemic, flying between the planets of our solar system to understand its scale, or operating on an injured soldier amidst a battle). However, transfer and retention must be better supported before VR could be widely adopted. As VR environments and real-world environments often differ greatly and share relatively few cues, context-change induced forgetting could lead to a lack of transfer and retention. Our study found the combination of “context crutch,” “desirable difficulties,” and mental reinstatement can help remedy these issues. Our findings show that rapid learning and transfer are possible, and one-week retention can be achieved when using VR, which may greatly increase the pedagogical value of VR for trainers and educators.

***Significance for health care: from medical to psychosocial therapy.*** Clinical health care involves much pedagogy—providers often need to teach patients and/or caregivers information that is important for the patients’ health. These may include medication regimen in elderly health, safety considerations for patients with narcolepsy, self-care tips for cancer patients, and therapeutic skills for patients with mental health conditions, etc. These pedagogy elements are subjected to the same constraints as other forms of learning and memory and can be enhanced in a similar way. First, most care-related information is delivered in the clinic, patients and caregivers must then recall the information beyond the clinic. Findings suggest that until the information has become generalised to day-to-day contexts, patients and caregivers may benefit from mentally reinstating the clinician’s office when planning out the care required for the day. Our study’s use of paired associates (i.e. English meanings paired with foreign words) made it particularly relevant for psychosocial therapy in which associative learning processes are involved.^1^ For example, in cognitive behavioural therapy (CBT), a patient suffering from specific phobia of dogs would learn a new association through exposure therapy in the clinic (dog 🡪 no fear) that inhibits the original, dysfunctional association (dog 🡪 fear). After forming therapeutic learning in one setting (e.g., clinician’s office), patients are challenged to recall these learning beyond the clinic (e.g., school, home, street) where context-change induced forgetting may impair recall. Our results suggest that leading patients to practice mental reinstatement of the learning context may help generalise therapeutic learning to contexts beyond the clinic. When the patient encounters a dog outside of the clinic, if the patient can mentally reinstate the learning context (the clinician’s office, face, and/or voice) they might be more likely to recall the non-fear association during the encounter. This would strengthen the therapeutic learning and make it more likely to be recalled in the future.

Second, our results suggest that future studies should examine whether switching contexts would reduce interference when multiple sets of care information are to be introduced at the same time, especially if patients and caregivers are instructed to mentally reinstate the learning context during recall. In telemedicine, distinctive virtual backgrounds behind clinicians maybe easy to implement; however, this may not be effective as the subjective sense of presence would likely be low (the patients are unlikely to see the virtual backgrounds behind the clinician as contexts that they are actually inhabiting). Instead, after providing one set of information, a clinician can request the patient or caregiver to take their devices and physically move to a distinctively different location before imparting another information set. For an in-person appointment, a clinician may teach one set of information in the examination room, then move with the patient or caregiver to a private waiting room that is distinctively different before teaching another information set. Although current medical facilities tend not to have highly distinctive rooms, further investigation may provide evidence to justify systemic incorporation of these distinctive contexts—especially when caring for clinical populations that have impaired learning and memory, such as patients with mild cognitive impairment or Alzheimer’s disease. When these solutions are not available, a possible work-around could be to teach the patient the information, then ask them to recall the information at home via video conferencing and provide correction should the recall be incorrect. This would enable patients or caregivers to re-learn the information in a context where future recall would likely occur (vis-à-vis “context crutch”). Moreover, patients or caregivers can physically move to a different room when recalling and relearning another set of information. Additionally, mental reinstatement of the learning context during recall should be considered until the information become generalised into multiple contexts of day-to-day life.

***D2. Comparison between Carpenter and Olson (2012) and the current study***

The current study’s Swahili word stimuli were based on the Carpenter and Olson (2012)^2^ stimuli, in order to enable a planned proximate comparison between their Test 1 after two exposures, to our study’s T2 after the second learning sessions. In Carpenter and Olson (2012) Experiment 2, participants were taught Swahili words across multiple test-learn cycles (Learn, Learn, Test1-Learn, Test2-Learn, Test3). In the English-Swahili condition, participants were cued by pictures or English translations to retrieve and type the Swahili spelling.

As the rapidity of acquisition was established by comparing the current study’s learning rate to that of Experiment 2 in Carpenter & Olson (2012), it is important to note that major differences in goals, designs, and methods existed between the two studies. The current study attempted to maximise learning, while Carpenter & Olson (2012) aimed to determine whether images were beneficial to foreign language learning. Moreover, the previous study’s Test 1 was the first test after two learning exposures; our T2 occurred after an additional test between the first and second exposure. Therefore, the difference in our results can be partly attributed to the additional retrieval practice. Moreover, unlike our T2 and T3, Carpenter & Olson’s Test 1 and Test 2 were not separated by an overnight delay.

Nonetheless, the comparison between our respective studies is of interest given that we used the same Swahili word list and both studies required foreign word retrieval in response to English word cues (this test-direction is rare in the extant literature).

**Appendix**

**I. Word List**

List of foreign words used in the experiment; some were modified to fit the experimental criteria. The foreign words were selected or modified so that each Chinyanja word and its Swahili counterpart: (a) did not begin with the same letter when transliterated to English; (b) differed in at least one syllable; and (c) did not differ in length greater than two syllables. From this list, each participant learnt 50 English words studied with a total of 80 foreign translations—10 in Swahili only, 10 in Chinyanja only, and 30 in both languages.

| **English** | **Swahili** | **Chinyanja** |  | **English** | **Swahili** | **Chinyanja** |
| --- | --- | --- | --- | --- | --- | --- |
| Apple | *m'boni* | *pitaya* |  | Giraffe | *twiga* | *n'swala* |
| Arrow | *m'shale* | *bano* |  | Gun | *bunduki* | *m'futi* |
| Axe | *shoka* | *tc'hado* |  | Hammock | *machela* | *kalichi* |
| Ball | *m'pira* | *bande* |  | Handbag | *m'koba* | *kachikwama* |
| Banana | *migomba* | *nt'hochi* |  | Hat | *kofia* | *chipewa* |
| Bath | *umwagaji* | *bafama* |  | Helmet | *chapeo* | *yamoto* |
| Bed | *kitanda* | *mp'hasa* |  | Horse | *farasi* | *hachi* |
| Beer | *pombe* | *mowa* |  | Key | *muhimu* | *fungulo* |
| Belt | *ukanda* | *lamba* |  | Knife | *kisu* | *m'peni* |
| Bicycle | *m'zunguko* | *n'jinga* |  | Lamp | *taa* | *get'si* |
| Bird | *n'dege* | *m'balame* |  | Lemon | *limau* | *indimu* |
| Boat | *mashua* | *b'wato* |  | Map | *ramani* | *kolowera* |
| Book | *kitabu* | *lemba* |  | Necklace | *kujitia* | *m'dendere* |
| Camel | *n'gamia* | *gamira* |  | Orange | *machungwa* | *lalanje* |
| Candle | *m'shumaa* | *n'yale* |  | Orca | *pomboo* | *mn'yanda* |
| Car | *magari* | *galimoto* |  | Pen | *kalamu* | *cholembera* |
| Carriage | *inasimamia* | *galetama* |  | Piano | *kinanda* | *mn'goli* |
| Cat | *yapaka* | *m'phaka* |  | Pig | *n'guruwe* | *k'humba* |
| Cattle | *fahali* | *n'gombe* |  | Pineapple | *mananasi* | *zinanazi* |
| Chair | *kiti* | *mipando* |  | Pot | *sufuria* | *nk'hali* |
| Cherry | *m'cheri* | *tundu* |  | Pumpkin | *malenge* | *d'zungu* |
| Coconut | *minazi* | *n'golema* |  | Rooster | *jogoo* | *tambala* |
| Cup | *kikombe* | *chifulu* |  | Sandal | *viatu* | *patapata* |
| Dog | *umbwa* | *galu* |  | Snake | *fira* | *n'joka* |
| Door | *m'lango* | *chit'seko* |  | Sword | *upanga* | *malupanga* |
| Dress | *mavazi* | *vaka* |  | Telescope | *darubini* | *owonera* |
| Firepit | *shimomoto* | *chiyoto* |  | Toilet | *choo* | *konyera* |
| Frog | *chura* | *mitundu* |  | Tomato | *nyanya* | *t'himati* |
| Games | *yamichezo* | *masewero* |  | Watch | *kuangalia* | *onatu* |

**II. Intrusion Coding**

Coding instructions for scorers to rate intrusions. Trials with a code of 3 and above were counted as intrusion trials.

| **Code** | **Description** |
| --- | --- |
| 0 | *Not an intrusion*   - correct word recall, or incorrect recall that bore no detectable resemblance to other learnt words |
| 1 | *Possible intrusion*, same language   - incorrectly recalled word that bore resemblance to a word learnt in the same language |
| 2 | *Sure intrusion, same language*   - incorrectly recalled word that nearly or exactly match another word learnt in the same language |
| 3 | *Possible intrusion, different language*   - incorrectly recalled word that bore some resemblance to a word learnt in the other language |
| 4 | *Sure intrusion with error, different language*   - incorrectly recalled word that bore high resemblance to a word learnt in the other language |
| 5 | *Sure intrusion without error, different language*   - incorrectly recalled word that was an exact match to a word learnt in the other language |
