# Supplementary material for: Enhancing learning and retention with distinctive virtual reality environments and mental context reinstatement
Source: NPJ Sci Learn. 2022 Dec 8;7:31. doi: 10.1038/s41539-022-00147-6 (PMC9732332; doi:10.1038/s41539-022-00147-6)
Supplement: Supplementary file 4 — Reporting Summary Checklist [file 41539_2022_147_MOESM4_ESM.pdf]

nature portfolio

Corresponding author(s): Jesse Rissman, Ph.D.

Last updated by author(s): Aug 17, 2022

Reporting Summary

Nature Portfolio wishes to improve the reproducibility of the work that we publish. This form provides structure for consistency and transparency in reporting. For further information on Nature Portfolio policies, see our Editorial Policies and the Editorial Policy Checklist.

Statistics

For all statistical analyses, confirm that the following items are present in the figure legend, table legend, main text, or Methods section.

n/a	Confirmed	
☐	☒	The exact sample size (*n*) for each experimental group/condition, given as a discrete number and unit of measurement
☐	☒	A statement on whether measurements were taken from distinct samples or whether the same sample was measured repeatedly
☐	☒	The statistical test(s) used AND whether they are one- or two-sided<br>*Only common tests should be described solely by name; describe more complex techniques in the Methods section.*
☐	☒	A description of all covariates tested
☐	☒	A description of any assumptions or corrections, such as tests of normality and adjustment for multiple comparisons
☐	☒	A full description of the statistical parameters including central tendency (e.g. means) or other basic estimates (e.g. regression coefficient) AND variation (e.g. standard deviation) or associated estimates of uncertainty (e.g. confidence intervals)
☐	☒	For null hypothesis testing, the test statistic (e.g. F, t, r) with confidence intervals, effect sizes, degrees of freedom and P value noted<br>*Give P values as exact values whenever suitable.*
☒	☐	For Bayesian analysis, information on the choice of priors and Markov chain Monte Carlo settings
☒	☐	For hierarchical and complex designs, identification of the appropriate level for tests and full reporting of outcomes
☐	☒	Estimates of effect sizes (e.g. Cohen's *d*, Pearson's *r*), indicating how they were calculated

Our web collection on statistics for biologists contains articles on many of the points above.

Software and code

Policy information about availability of computer code

Data collection	OpenSimulator, Firestorm Viewer for OpenSim, PsychoPy, Audacity, QuickTime Player, GoogleVoice
Data analysis	MATLAB, SPSS, FSL, AFNI, EXCEL

For manuscripts utilizing custom algorithms or software that are central to the research but not yet described in published literature, software must be made available to editors and reviewers. We strongly encourage code deposition in a community repository (e.g. GitHub). See the Nature Portfolio guidelines for submitting code & software for further information.

Data

Policy information about availability of data

All manuscripts must include a data availability statement. This statement should provide the following information, where applicable:

- Accession codes, unique identifiers, or web links for publicly available datasets
- A description of any restrictions on data availability
- For clinical datasets or third party data, please ensure that the statement adheres to our policy

All de-identified data are available upon request. The MATLAB scripts used for fMRI daa processing and statistical analysis are available from the corresponding author, upon reasonable request for purposes of reproducing or extending the analysis.

Human research participants

Policy information about studies involving human research participants and Sex and Gender in Research.

Reporting on sex and gender	As the literature that forms the theoretical bases of our hypotheses did not speak to sex/gender effects, neither factored into the study design. However, we strived to recruit a relatively balanced sample; based on self-reported sex, our sample consisted of 26 females and 22 males in the behavioural experiment, and 12 females and 10 males in the fMRI experiment (see Supplemental Table 1). Our sample is inadequately powered to conduct robust analyses of potential sex-related effects.
Population characteristics	Population Characteristics: As it was not pertinent to a priori hypotheses based on previous literature, we did not collect medical, mental health, and medication history except that which related to the screening criteria. An initial screening question that combined a variety of factors was asked in a manner such that participants could answer Yes or No to the combined question without providing any specifics, so as to minimise privacy concerns ("Have you used SecondLife or OpenSim for more than 5 hours, have vision and audition difficulties that cannot be corrected with devices such as glasses or hearing aids, any diagnosis of learning disabilities or substance dependence; or are taking psychotropic medications?").
Recruitment	Participants were recruited through flyers posted around the campus of University of California, Los Angeles (UCLA) and social media advertisements targeting the same geographical area. Participants were tested individually, and they received course credit or were compensated monetarily ($20 per hour for fMRI procedures, $10 per hour for non-fMRI procedures).
Ethics oversight	The Institutional Review Board of University of California, Los Angeles (UCLA).

Note that full information on the approval of the study protocol must also be provided in the manuscript.

Field-specific reporting

Please select the one below that is the best fit for your research. If you are not sure, read the appropriate sections before making your selection.

☐ Life sciences ☒ Behavioural & social sciences ☐ Ecological, evolutionary & environmental sciences

For a reference copy of the document with all sections, see nature.com/documents/nr-reporting-summary-flat.pdf

Behavioural & social sciences study design

All studies must disclose on these points even when the disclosure is negative.

Study description	Investigation of contextual effects on foreign vocabulary learning using quantitative data (between-subject design in the behavioural experiment, within-subject design in the fMRI experiment).
Research sample	Participants were adults who resided in the Los Angeles community (ages 18-27), the majority of whom were students attending university in the UCLA area. 38 out of 70 of the participants (54%) were female.
Sampling strategy	Participants were recruited and enrolled based on convenience sampling. Sample size for the behavioural experiment (24 participants per group, 48 total) was determined a priori based on resource and time constraints (i.e. each participant contributed nearly 10 hours of their time across 3 sessions, and scoring of the verbal response data was time-consuming), as well as a previous study with a design most similar to ours (Carpenter & Olson, 2012, Experiment 2, n=24). Although a dearth of comparable studies made it hard to estimate our expected effect size, we chose a per-group sample size that was typical at the time for task-based fMRI studies, with the idea that if our behavioural experiment showed robust effects, then our planned follow-up fMRI experiment would be viable. We intended to acquire data from 24 participants in our fMRI study, but one dataset could not be collected due to a late cancellation of a participant, and one fMRI participant fell asleep during the recall task and was excluded from analyses.
Data collection	In the behavioural experiment, all Day 1 and Day 2 procedures occurred in the laboratory's VR room. This is also true for the fMRI experiment except for the non-VR recall (T4) test, which occurred in the MRI scanner. For all participants, the surprise, one-week delay recall (T5) test occurred over the telephone. All data were collected digitally, these included digital recording of verbal responses, survey data, and fMRI data; no pencil and paper forms were used for data collection.<br><br>In the laboratory, procedures were conducted in a room with desktop VR computer equipment. The room's window was blacked out to prevent distractions and to ensure all participants experienced the VR in the same lighting regardless of time of day. The experimenter was present during all procedures. The participant and experimenter each sat at their own desk with a cubical separator between them. After informed consent and general instructions, the participant and experimenter both 'entered' the desktop VR environment and communicated with one another via VoIP functionality using over-the-ear headphones with built-in microphones, and navigated and interacted with the VR using keyboard and mouse. In the practice world, the participant received in-VR instructions on the two experimental tasks (context encoding task and language encoding task) and practiced the tasks under experimenter supervision. The participant then performed each task in the assigned context, while the experimenter monitored them to ensure that instructions were followed correctly. Verbal response were recorded on the experimenter's computer.<br><br>In the non-VR recall test, the experimenter provided instructions to the participant using a slideshow presentation. Then the

computerised verbal recall task was presented and verbal responses were recorded. In the fMRI experiment, the computerised verbal recall task took place while the participant was in the scanner, and the experimenter monitored the participant from the MRI control room; a noise-cancelling microphone was used to collect verbal responses.

In the surprise one-week delay recall task, the experimenter phoned the participant at a pre-scheduled time. The call was made via a laboratory computer using VoIP, and the recording was saved on the computer with participant consent.

For additional details, please see the methods and supplemental methods where data collection procedures were reported extensively.

Timing

Data was collected between March of 2015 and February 2017.

Data exclusions

Trial by trial data exclusion criteria are detailed in the methods section. Behavioural experiment data from an additional 13 people were acquired but excluded from analyses: five did not complete procedure due to technical difficulties, three withdrew due to motion sickness during their desktop-VR experience, three did not return for Day 2 procedures, and two were excluded for not following instructions. fMRI experiment data from one additional person was acquired but excluded from analyses, for this individual reported falling asleep during procedure.

Non-participation

As mentioned in the data exclusion section, three consented participants withdrew from the behavioural experiment due to motion sickness during their desktop-VR experience; three additional participants did not return for Day 2 procedures. This study shared recruitment efforts with three other VR-based experiments, for which a total of 1,394 screening surveys were submitted, amongst them, 282 were eligible to participate (most were ineligible due to the specific language requirements, where participants needed to be mono-lingual in the behavioural experiment, and exactly dual-lingual in the fMRI experiment). Amongst the 282, 83 chose to participate in this study over the other on-going studies, which were shorter and involved less visits.

Randomization

In the behavioural experiment, participants were randomly assigned to the single- or dual-context group. No group assignment occurred for the fMRI experiment which was a within-subject design with only dual-context participants.

Reporting for specific materials, systems and methods

We require information from authors about some types of materials, experimental systems and methods used in many studies. Here, indicate whether each material, system or method listed is relevant to your study. If you are not sure if a list item applies to your research, read the appropriate section before selecting a response.

Materials & experimental systems

n/a	Involved in the study
☒	☐ Antibodies
☒	☐ Eukaryotic cell lines
☒	☐ Palaeontology and archaeology
☒	☐ Animals and other organisms
☒	☐ Clinical data
☒	☐ Dual use research of concern

Methods

n/a	Involved in the study
☒	☐ ChIP-seq
☒	☐ Flow cytometry
☐	☒ MRI-based neuroimaging

Magnetic resonance imaging

Experimental design

Design type

Event-related design

Design specifications

In the fMRI experiment, the non-VR recall test (T4) was conducted in the MRI scanner. A total of 80 trials were presented to each participant in a single MRI session. Trials were presented in 10 blocks of 8 trials each. Each trial was 38 seconds in duration, with 5 seconds of active baseline tasks between trials.

Behavioral performance measures

The primary measure was verbal recall score. After 8 seconds of covert recall, the participant said aloud the foreign word they had retrieved; these were recorded and scored offline to produce the recall scores (awarding partial credit for getting some syllables correct) reported in the manuscript.

Acquisition

Imaging type(s)

Functional and structural

Field strength

3.0 Tesla

Sequence & imaging parameters

fMRI data were collected with a Siemens 3.0 Tesla Magnetom PrismaFit scanner at the UCLA Ahmanson-Lovelace Brain Mapping Center, using a 64-channel head coil. Functional data were acquired using T2*-weighted simultaneous multislice echoplanar imaging (EPI) sequences (TR = 1.0 s; TE = 30 ms; flip angle = 52°; FoV = 20.8 cm; multiband acceleration factor = 5; 65 oblique axial slices; voxel resolution 2 x 2 x 2 mm). Each of the 10 runs consisted of 330

volumes and included 8 trials of the task (we did not discard initial volumes as the version of Syngo software did not begin recording until T1 stabilised). Additionally, a T1-weighted structural MRI [axial magnetisation-prepared rapid gradient-echo (MPRAGE), 0.8mm3] was obtained for spatial registration of the functional data.

Area of acquisition: Whole brain scan

Diffusion MRI: ☐ Used ☒ Not used

Preprocessing

Preprocessing software: Functional data were pre-processed without spatial smoothing, pre-whitening, nor B0 unwarping using the FMRI Software Library (FSL) 5.0.4 and Advanced Normalisation Tools (ANTS) 2.0. FSL Brain Extraction Tool (BET2) was used to perform brain extraction.

Normalization: Images were registered to standard Montreal Neurological Institute (MNI) template using FMRIB's Linear Image Registration Tool (FLIRT).

Normalization template: MNI152

Noise and artifact removal: FSL FEAT was used to apply a high-pass temporal filter (128 Hz). Timeseries alignment and motion correction were performed in FSL Motion Correction FLIRT (MCFLIRT) and ANTS.

Volume censoring: No volumes were censored.

Statistical modeling & inference

Model type and settings: For the searchlight multi-voxel pattern analysis (SL-MVPA), we used a 3-voxel radius to create searchlight spheres, and using the voxel activity values within each sphere as features, we performed leave-one-trial-out cross-validation with a support vector machine (SVM) classifier. For the representational similarity analysis (RSA), we used MATLAB to conduct Pearson correlations between vectorised representations of voxel activity.

Effect(s) tested: Resultant SL-MVPA maps were used to select voxels for the RSA. Specifically, we sorted voxel indices as a function of their assigned classification accuracy and used the top 2000 voxels as our RSA mask for each participant. RSA was used to determine fidelity of learning-context mental reinstatement during each recall trial. Based on each participant's RSA data, we subdivided their trials into high-fidelity and low-fidelity reinstatement and analysed the verbal recall data for each trial type.

Specify type of analysis: ☒ Whole brain ☐ ROI-based ☐ Both

Statistic type for inference (See Eklund et al. 2016): RSA values from each trial were only used to subdivide trials into high-fidelity and low-fidelity reinstatement, so no statistical tests were performed directly on the RSA data. Repeated-measures ANOVA was used to assess the effects of reinstatement fidelity on verbal recall performance.

Correction: As fMRI data were only used to separate behavioural data into discrete categories, multiple comparison correction was not necessary.

Models & analysis

n/a	Involved in the study
☒	☐ Functional and/or effective connectivity
☒	☐ Graph analysis
☐	☒ Multivariate modeling or predictive analysis

Multivariate modeling and predictive analysis: For the SL-MVPA, we created a series of 3-voxel radius ROIs surrounding each voxel in the whole-brain mask using the adj_sphere.m function (Princeton MVPA Toolbox). ROIs whose extent would have fallen outside the whole-brain mask were truncated to fit within the mask, creating uneven feature set sizes as a function of the voxel's proximity to the borders of the mask. Within each ROI, we employed an SVM classifier (C-SVC; polynomial kernel; c=1; LIBSVM) in a leave-one-trial-out cross-validation. Accuracy was computed as the number of trial types correctly output by the classifier divided by the total number of trials used in the cross-validation. The resultant accuracy value was saved to the central voxel that was used to create the ROI, and these values were used to select the top 2000 voxels for each participant for subsequent use in the RSA. For the RSA, we used MATLAB to compute the Pearson correlations between vectorised representations of voxel activity.
