# Supplementary material for: Enhancing learning and retention with distinctive virtual reality environments and mental context reinstatement
Source: NPJ Sci Learn. 2022 Dec 8;7:31. doi: 10.1038/s41539-022-00147-6 (PMC9732332; doi:10.1038/s41539-022-00147-6)
Supplement: Supplementary file 5 — Supplementary Videos Legends [file 41539_2022_147_MOESM5_ESM.docx]

**Video 1 | Demonstration of Language Encoding Task.** An example of the Language Encoding trials that occurred in Fairyland Garden. Participant would verbally repeat after the "teacher" each time the foreign word was heard.

**Video 2 | Day 1 Instructional Video for Participant.** On Day 1, after informed consent and general instructions, participants “entered” the introductory VR environment (Figure 1.a1). Therein, participants first familiarised themselves with the navigational controls. They then received instructions for the context encoding and language encoding tasks by watching this video on a screen (an object within the VR). Then, they practiced the two tasks (Figure 1a.2) under the supervision of an experimenter, who provided corrective feedback to ensure that participants had proper understanding of the tasks. The experimenter was present in the same physical room and in the same VR environment as an avatar, and communicated with participants via in-world VoIP connection.
